# Mutation of *SELF-PRUNING* homologs in cotton promotes short-branching plant architecture

**DOI:** 10.1093/jxb/ery093

**Published:** 2018-03-14

**Authors:** Zhanfeng Si, Hui Liu, Jiankun Zhu, Jiedan Chen, Qiong Wang, Lei Fang, Fengkai Gao, Yue Tian, Yali Chen, Lijing Chang, Bingliang Liu, Zegang Han, Baoliang Zhou, Yan Hu, Xianzhong Huang, Tianzhen Zhang

**Affiliations:** 1 Agronomy Department, College of Agriculture and Biotechnology, Zhejiang University, Hangzhou, China; 2 Special Plant Genomics Laboratory, College of Life Sciences, University of Shihezi, Shihezi, Xinjiang, China; 3 State Key Laboratory of Crop Genetics and Germplasm Enhancement, Cotton Hybrid R & D Engineering Center (the Ministry of Education), College of Agriculture, Nanjing Agricultural University, Nanjing, China

**Keywords:** *cl*_*1*_^*b*^, *cl*_*1*_, cotton *SELF-PRUNING* gene, flowering, plant architecture, yield increase

## Abstract

In cotton, the formation of fruiting branches affects both plant architecture and fiber yield. Here, we report map-based cloning of the axillary flowering mutation gene (*GbAF*) that causes bolls to be borne directly on the main plant stem in *Gossypium barbadense,* and of the clustered boll mutation gene (*cl*_*1*_) in *G. hirsutum*. Both mutant alleles were found to represent point mutations at the *Cl*_*1*_ locus. Therefore, we propose that the *GbAF* mutation be referred to as *cl*_*1*_^*b*^. These *Cl*_*1*_ loci correspond to homologs of tomato *SELF-PRUNING* (*SP*), i.e. *Gossypium* spp*. SP* (*GoSP*) genes. In tetraploid cottons, single monogenic mutation of either duplicate *GoSP* gene (one in the A and one in the D subgenome) is associated with the axillary cluster flowering phenotype, although the shoot-indeterminate state of the inflorescence is maintained. By contrast, silencing of both *GoSPs* leads to the termination of flowering or determinate plants. The architecture of axillary flowering cotton allows higher planting density, contributing to increased fiber yield. Taken together the results provide new insights into the underlying mechanism of branching in cotton species, and characterization of *GoSP* genes may promote the development of compact cultivars to increase global cotton production.

## Teaser

We characterize *GoSP* genes underlying the development of cotton plants with short branches and clustered bolls, a phenotype that allows higher planting density and promotes increased fiber yield per acre.

## Introduction

In flowering plants, shoot apical meristems (SAMs) generate aerial organs such as leaves, stems, branches, and floral structures. SAMs contain a population of pluripotent stem cells that divide to generate new cells for the continual formation of new tissues and organs (Bowman and Eshed, 2000; Barton, 2010). *TERMINAL FLOWER1* (*TFL1*) in Arabidopsis (Shannon and Meeks-Wagner, 1991; Bradley *et al.*, 1997), *CENTRORADIALIS* (*CEN*) in *Antirrhinum* (Bradley *et al.*, 1996), and *SELF-PRUNING* (*SP*) in tomato (*Solanum lycopersicum*) (Pnueli *et al.*, 1998; Carmel-Goren *et al.*, 2003) all function to maintain the indeterminate state of the SAM. Therefore, these genes are key regulators of plant architecture, as they control flowering transition and the fate of SAMs. The *CEN/TFL1/SP* (*CETS*) gene family shares homology with the phosphatidylethanolamine-binding protein (PEBP) gene family, the latter consisting of three main subfamilies: *MOTHER OF FT AND TFL1* (*MFT*), *FLOWERING LOCUS T* (*FT*), and *TFL1* (Chardon and Damerval, 2005; Hedman *et al.*, 2009). FT protein, commonly known as florigen, is transported from leaves to the shoot apex. There, FT interacts with receptor 14-3-3 proteins and binds to the basic leucine zipper (bZIP) transcription factor FD (Abe *et al.*, 2005; Wigge *et al.*, 2005; Corbesier and Coupland, 2006; Tamaki *et al.*, 2007) to activate floral-meristem identity genes such as *APETALA1* (*AP1*) (Abe *et al.*, 2005; Wigge *et al.*, 2005) and *LEAFY* (*LFY*) (Schultz and Haughn, 1991). *FT* and *TFL1* encode related proteins with similarity to human Raf kinase inhibitor (Kobayashi *et al.*, 1999), but FT homologs promote flowering, whereas TFL1 homologs repress flowering and maintain meristems in an indeterminate state.

Natural variations of *SP* have been exploited in plant breeding to increase yield in many crops, including soybean (Liu *et al.*, 2010), grapes (Fernandez *et al.*, 2010), barley (Comadran *et al.*, 2012), roses (Iwata *et al.*, 2012), strawberries (Iwata *et al.*, 2012), and tomato (Park *et al.*, 2014; Soyk *et al.*, 2017). Tomato cultivation was revolutionized following the discovery of an *SP* mutant, which is considered the single most important genetic trait for modern tomato agriculture (Pnueli *et al.*, 1998). That tomato *SP* mutation led to determinate plants with a burst of flowering and synchronized fruit ripening (Soyk *et al.*, 2017). Tomato productivity has been further optimized by exploiting combinations of select mutations comprising multiple florigen pathway components (Park *et al.*, 2014). For example, tomato plants heterozygous for *SFT* loss-of-function alleles have a yield increase up to 60% (Krieger *et al.*, 2010).

**Fig. 1.**
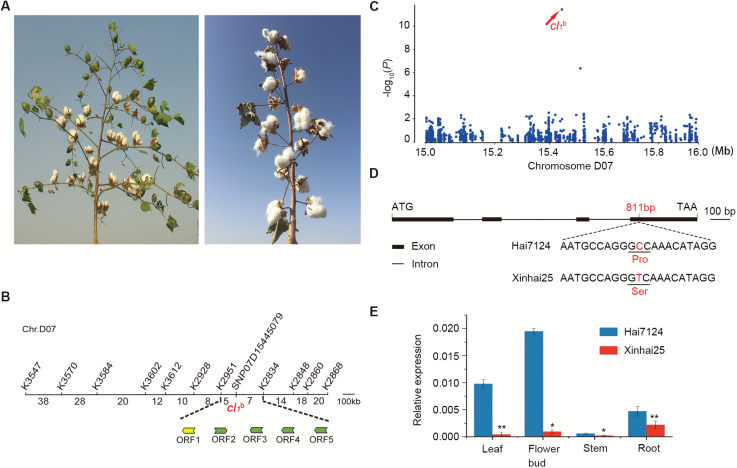
Fine mapping and cloning of axillary flowering. (A) The long-branching WT phenotype (left) and the GbAF phenotype (right) in *G. barbadense*. Left, a fruiting branch typical of WT cotton; right, the GbAF phenotype, typified by buds that are borne directly on the main stem with more than one bud or axillary flower. (B) Fine mapping of *GbAF* with indel and SNP primers. (C) Manhattan plot for the GWAS. The SNPs were located within the strongest association signal (arrow indicated). (D) The mutated SNP led to an amino acid substitution from aspartic proline to serine at position 811 bp of *GbSP*. (E) qRT-PCR analysis of *GbSP* gene expression in WT Hai7124 and GbAF-phenotype Xinhai25 leaf, flower bud, stem, and root tissue. Data are averages of three independent biological replicates ±SEM. Experimental gene expression is normalized to *GhHIS3* (AF024716). **P<0.01, *P<0.05 (Student’s *t*-test). (This figure is available in color at *JXB* online.)

Together, the cultivated allotetraploid upland cotton (AD)_1_ (*Gossypium hirsutum* L.) and the extra-long staple (ELS) cotton (AD)_2_ (*G. barbadense* L.) are the largest source of renewable textile fiber worldwide. Wild-type allotetraploid cotton plants continuously produce new shoots and inflorescences from shoot meristems, resulting in indeterminate plant architecture. The fruiting branch (or sympodium) is a major determinant of cotton plant architecture and is therefore agronomically important. Cotton first produces vegetative branches (or monopodia), which in turn bear fruiting branches, and then typically produces fruiting branches on the main stem in a descending order, giving rise to a pyramidal plant architecture (Fig. 1A; Supplementary Fig. S1 at *JXB* online). There are two kinds of fruiting branch mutants in cotton. In contrast to the ELS Pima/Egyptian cotton, all modern ELS cultivars developed and grown in China display the axillary flowering phenotype (herein referred to as the GbAF phenotype), alternately termed nulliplex-type branching (Chen *et al.*, 2015). The GbAF phenotype is characterized a lack of fruit branches, which causes one or more clustered flowers with elongated pedicels to be attached directly to the main stem, and therefore creates a compact architecture that allows high-density planting for increased cotton yield (Silow, 1946). The GbAF character is the single most important genetic trait in relation to cotton production as it not only increases lint yield but also facilitates mechanical harvesting. Thus, the GbAF phenotype has been exploited for cotton cultivar development in China.

The clustered boll phenotype exemplified by *G. hirsutum* (herein referred to as the GhCB phenotype) represents another cotton plant architecture characterized by short branches in which the fruit branches are reduced to a single developed internode, and two or more flowers are borne at their apex. When Silow (1946) first identified a homoeologous linkage group for the cluster fruiting habit, the associated loci were referred to as *cl*_*1*_ in the D subgenome in *G. hirsutum* and *cl*_*2*_ in the A subgenome in *G. barbadense* (Silow, 1946). Thus, the GhCB phenotype was known to be associated with a duplicated gene, which remained to be cloned.

Here, we describe the cloning of *GbAF* and *cl*_*1*_. We found these genes to be allelic variations of the long-sympodium *Cl* locus and orthologs of tomato *SP*, and therefore denoted them as *Gossypium* spp*. SP* genes (*GoSPs*). Our results demonstrate that mutations in *GoSPs* confer a compact growth phenotype that may be exploited for improved cotton cultivation and mechanical harvesting.

## Materials and methods

### Plant material

*Gossypium hirsutum* acc. TM-1 with normal branches was obtained from the Southern Plains Agricultural Research Center, USDA-ARS, College Station, TX, USA. *Gossypium barbadense* cv. Xinhai 25, with monopodial branches, planted in Xinjiang, the north-western cotton growing region of China, was developed by the Institute of Agricultural Sciences Agricultural Division 1, the Xinjiang Production and Construction Corps, Alaer, Xinjiang. *Gossypium barbadense* cv. Hai7124, with normal branches, grown extensively in China, was the offspring of a selected individual in studies of inheritance of resistance to *Verticillium dahlia*. *Gossypium hirsutum* acc. T582 is a line with multiple markers, including cluster bolls, glandless stem, cup leaf, virescent, and frego bract, but with the same genetic background as the genetic standard line of upland cotton, TM-1. T582 was kindly made available by the Southern Plains Agricultural Research Center. The 229 *G. barbadense* cultivars used are listed in Supplementary Table S1.

### Genome-wide association study analysis

Paired-end reads were aligned against the cotton reference genome (*G. hirsutum* acc. TM-1) using BWA-MEM (version 0.7.12) (Li and Durbin, 2009). SAMtools (version 0.1.19) (Li *et al.*, 2009) was used to convert mapping results to the bam format, and duplicated reads were filtered with the help of the Picard package (version 1.128). Only the uniquely aligned reads that were mapped to unique locations in the reference genome were retained (SAMtools flags −F 256 −f 3 −q 20). The threshold of single nucleotide polymorphism (SNP) calling was set to 20 for both base quality and mapping quality. SNP identification and genotype calling were performed based on the outputs from mpileup of the SAMtools software package. Only the non-singleton SNPs, defined as those where more than two accessions demonstrate the presence of the alternative alleles, were retained. Unreliable SNP sites were then filtered out, the candidate SNP loci were required to be bi-allelic, and all the singleton SNPs were excluded. We required that the common SNPs had a minor allele frequency (MAF) of more than 5%. We only analysed the SNPs that were located in the 26 pseudomolecules of the TM-1 assembly, and the SNPs in the small scaffolds (Zhang *et al.*, 2015) were removed. The EMMAX (Efficient Mixed-Model Association eXpedited) program was used to perform a large-scale genome-wide association study (GWAS). Association analysis was performed using the EMMAX software package. The significance threshold was determined by modified Bonferroni correction (Genetic type 1 Error Calculator (GEC) version 0.2) (Fang *et al.*, 2017).

### Genomic DNA extraction and sequencing pool construction

We extracted DNAs from 28 cluster boll individuals from (T582×TM-1)BC_1_ progeny and bulked these in an equal ratio to generate a ‘mutant type’ pool to conduct whole-genome resequencing with T582 parents. A total of 221.6 Gb of short (101-bp) paired-end reads was generated, including 94.7 Gb reads (37.9-fold genome coverage) for T582 and an average of 25.4 Gb, ranging from ~17.9 to 34.4 Gb (~7.2- to 13.8-fold genome coverage) for five bulks for different mutant traits (virescent-1 (v_1_), cup leaf (cu), glandless-1 (gl_1_), frego bract (fg) and cluster-1 (cl_1_)) (Zhu *et al.*, 2017). These reads were trimmed with Sickle software and then aligned to the TM-1 reference genome (Zhang *et al.*, 2015). A total of 347629 SNPs were identified between the two parents, TM-1 and T582. The reads from the mutant bulked pools were then aligned in order to calculate the ratio of the number of reads corresponding to the two parental genomes. In principle, the causative genomic regions should be in accord with all recessive extreme type BC_1_ plants; otherwise we would regard them as unrelated genomic regions.

In order to identify the genomic regions related to the T582 genotype, the allele frequencies and the statistical significance of the allele frequency difference (Fisher’s exact test) were evaluated in a block with a minimum reading depth of 20 consecutive SNPs across the whole genome. In order to avoid a significant difference in terms of the sequencing and the alignment error, an average –log_10_(*P*) value was applied. Genomic regions with an average –log_10_(*P*) value >2 were identified and considered as the candidate regions (Abe *et al.*, 2012; Takagi *et al.*, 2013; Takagi *et al.*, 2015).

### qRT-PCR analysis

RNA was extracted from different tissues from plants with different branch types using Plant RNA Rapid Extraction Kit (Molfarming, Nanjing, China). Total RNA was reversed to cDNA using a HiScript II Reverse Transcriptase Kit (Vazyme, Nanjing, China). qRT-PCR was carried out on an Applied Biosystems 7500 Fast Real-Time PCR System (Life Technologies, Carlsbad, CA, USA) in a 20 μl volume containing 100 ng of cDNA, 4 pM of each primer, and 10 μl of AceQ qPCR SYBR Green Master Mix (Vazyme) according to the manufacturer’s protocol. The PCR conditions were as follows: primary denaturation at 95 °C for 20 s followed by 40 amplification cycles of 3 s at 95 °C, and 30 s at 60 °C. Melting curve analysis was performed to ensure there was no primer-dimer formation. Information on the qRT-PCR primers for gene expression analysis is listed in Supplementary Table S2. Three replicate assays were performed with independently isolated RNAs, and each RT reaction was loaded in triplicate. Relative gene expression levels were calculated using the 2^–ΔΔ*C*t^ method (Livak and Schmittgen, 2001). Statistical analysis of the number of rosette leaves was performed using one-way ANOVA (Duncan’s multiple range tests). Statistically significant differences (*P*<0.05) are indicated by different lower-case letters.

### Virus-induced gene silencing assay

For the virus-induced gene silencing (VIGS) assay, transformed *Agrobacterium* colonies containing pTRV1 and pTRV2-GoSP were grown overnight at 28 °C in an antibiotic selection medium containing 50 mg 1^–1^ each of rifampicin and kanamycin. *Agrobacterium* cells were collected and resuspended in infiltration medium (10 mM MgCl_2_, 10 mM MES and 200 mM acetosyringone), adjusted to an OD_600_ 0.5. *Agrobacterium* strains containing TRV1 and TRV2 vectors were mixed at a ratio of 1:1. Seedlings with mature cotyledons but without a visible rosette leaf (7 d after germination) were infiltrated by inserting the *Agrobacterium* suspension into the cotyledons via a syringe. The plants were grown in pots at 25 °C in a growth chamber under a 16 h light–8 h dark cycle with 60% humidity (Gao *et al.*, 2011).

### Plasmids construction and transformation of Arabidopsis

Complete open reading frame cDNA of four *GoSP*s was amplified by RT-PCR using gene specific primers (Supplementary Table S3), and separately subcloned into pMD19-T vector (TaKaRa, Dalian, China). All constructs confirmed by sequence analysis were then transferred into pCAMBIA 2300-35S-OCS binary vectors to construct *35S:GoSPs* under control of the CaMV*35S* promoter.

The seeds of Arabidopsis mutant *tfl1-1*, obtained from the Arabidopsis Biology Resources Center (ABRC, Columbus, OH, USA), were sown in pots containing peat soil and vermiculite (1:1) and cultivated under long day conditions in the phytotron (22/17 °C day/night temperatures, 200 µmol m^–2^ s^–2^). Transgenic plants were generated by the floral-dip method (Clough and Bent, 1998). Transgenic plants were selected on half-strength MS culture medium containing 50 µg ml^–1^ kanamycin. The kanamycin-resistant plants were transplanted and subsequently monitored for flowering using non-transgenic wild-type (WT) seedling as a control. Statistical analysis of the number of rosette leaves was performed using one-way ANOVA (Duncan’s multiple range tests). Statistically significant differences (*P*< 0.05) are indicated by different lower-case letters.

### Subcellular location analysis

To generate the GoSP–green fluorescent protein (GFP) fusion protein construct, the 0.7 kb GFP coding sequence was first ligated into the *Xba*I and *Sal*I sites of the binary vector pCAMBIA2300-35S-OCS to construct *35S:GFP*. The coding regions of *GoSPs* without stop codon were amplified by PCR and inserted into the *Kpn*I and *Xba*I sites of the *35S:GFP* vector to generate *35S:GoSP-GFP* in-frame fusion. The transient expression assays in tobacco were performed according to a previous method (Li *et al.*, 2015). The *A. tumefaciens* strain GV3101 carrying *35S:GoSP-GFP* was grown at 28 °C in LB medium with kanamycin and rifampicin to an OD_600_ of ~0.5–0.6. The agrobacterial cells were centrifuged and resuspended in 10 mM MgCl_2_, 10 mM MES–KOH (pH5.7) and 150 μM acetosyringone to an OD_600_ of 0.5. The agrobacterial cells were left to stand for 3 h at room temperature and then infiltrated into the abaxial surface of 3-week-old tobacco (*Nicotiana benthamiana*) leaves with syringes. After ~3–5 d the infiltrated leaves were selected by detection of GFP fluorescence using a confocal microscope (LSM510; Zeiss, Jena, Germany).

### Yeast two-hybrid assays

Yeast two-hybrid assays were performed using the BD Matchmaker system (Clontech, Mountain View, CA, USA). The coding sequences of *GoSPs* and *GhFT* were amplified and cloned into pGBKT7 (Clontech) to produce BD-GoSPs and BD-GhFT, respectively, and the coding sequences of *GhFD* were amplified by PCR and cloned into pGADT7 to produce AD-GhFD. The resulting vectors of AD fusions and BD fusions were co-transformed into the yeast strain AH109, which has chromosomally integrated reporter genes *LacZ* and *His* under the control of the *GAL1* promoter. The transformed cells were selected on medium lacking histidine, tryptophan, and leucine (SD−His/−Trp/−Leu/−Ade) supplemented with 30 mM 3-amino-1,2,4-triazole (3-AT).

### Bimolecular fluorescence complementation assay

To generate the constructs for bimolecular fluorescence complementation (BiFC) assays, coding regions of *GhFT*, *GhSP*, *GbSP*, *Ghsp*, *Gbsp*, and *GhFD* were separately amplified and cloned into the pDONRZeo vector (Invitrogen) for fusion with the N-terminus of PVYNE or the C-terminus of PSCYCE vectors (Waadt *et al.*, 2008) by the LR reaction. The resulting plasmids were introduced into *A. tumefaciens* GV3101 cells, which were then infiltrated into 4-week-old *N. benthamiana* leaves for transient expression. In brief, the *A. tumefaciens* strains harboring BiFC cassettes were resuspended in infiltration buffer (10 mM MES pH 5.6, 150 μM acetosyringone, and 10 mM MgCl_2_) at an OD_600_ of 0.8. The resuspensions containing each BiFC construct were mixed at a 1:1 ratio and incubated for 3 h at room temperature, before being infiltrated into the abaxial surface of 4-week-old tobacco leaves with a syringe. Two days after infiltration, the tobacco leaves were imaged under a Zeiss LSM 710 confocal microscope. Primers used to construct the BiFC plasmids are shown in Supplementary Table S3.

### Bimolecular luciferase complementation assays

For bimolecular luciferase complementation assays, coding regions of *GhFD*, *GhSP*, Ghsp^73Asn^, *GbSP*, and Gbsp^113Ser^ were cloned into pDnor221 by BP reactions. The primers used to amplify coding regions of *GoSP* genes are listed in Supplementary Table S3. An entry clone of *GhFD* was introduced into a *35S:NLuc* plasmid to generate the *35S:GhFD-NLuc* fusion construct by the LR reaction, and entry clones of *GhSP*, Ghsp^73Asn^, *GbSP*, and Gbsp^113Ser^ were introduced into *35S:CLuc* plasmid by LR reactions to generate *35S:CLuc-GoSPs* fusion constructs. *35S:LUC*, *35S:NLuc*, *35S:CLuc*, and the expression constructs were mobilized into *A. tumefaciens* GV3101 for *N. benthamiana* transformation.

*Agrobacterium tumefaciens* bacteria containing the indicated constructs were transiently transformed into the abxial leaves of *N. benthamiana* as described previously (Chen *et al.*, 2008), and samples were analysed 2 d after inoculation. Before measurement, leaves injected with *A. tumefaciens* were evenly sprayed with 1 mM Beetle Luciferin, Potassium Salts (Promega), and were then placed in the dark for 3 min. An *in vivo* plant imaging system (LB 985 NightSHADE, Berthold Technologies, Germany) was used to capture the LUC image. There were at least three biological replicates with independent plants for each assay.

### Phylogenetic analysis

Alignment of the amino acid sequences of the *CENTRORADIALIS* families in cotton and other species was performed using the CLUSTALX program (Thompson *et al.*, 1997). MEGA5.1 (Tamura *et al.*, 2011) was used for a phylogeny reconstruction analysis using the Neighbor-Joining method and Poisson correction distance model. Bootstrap analysis was performed to estimate nodal support on the basis of 1000 resamplings.

### Folding prediction of the GoSP proteins

The structure of the cotton SP proteins were predicted against the *Antirrhinum* CEN protein structure (PDB ID: 1QOU) using Deep View/Swiss-pdb viewer 4.0.1 as described previously (Guex and Peitsch, 1997). The 3D structures of GoSP proteins were derived from the pre-structured proteins using SWISS-MODEL and compared with each other. Alignment diversity color was applied.

## Results

### Fine mapping and cloning of the *GoSP* allele *GbAF*

We used both map-based cloning and GWAS techniques to clone *GbAF*. A mapping population produced by a cross between *G. barbadense* cv. Xinhai 25 (Fig. 1A; Supplementary Fig. S1A) and *G. hirsutum* acc. TM-1, containing *GbAF* and its corresponding WT allele, respectively, was used to map *GbAF*. All (TM-1×Xinhai 25)F_1_ plants possessed normal long fruit branches, and F_2_ plants segregated into 744 WT and 254 GbAF phenotypes. This phenotype segregation closely fitted a 3:1 ratio (*χ*^*2*^_3:1_=0.0855), suggesting a recessive monogenic inheritance pattern (Supplementary Table S4). Using this F_2_, *GbAF* was mapped to chr.D07 with microsatellite (SSR) markers (Supplementary Table S5; Supplementary Fig. S2). *GbAF* appeared to be a novel gene that differed from the clustered boll-2 (*cl*_*2*_), which was mapped to chr.A07. The *cl*_*2*_ mutant gene was first identified in *G. barbadense* Pima cotton in 1924 and initially named short branch (Kearney, 1930), but later renamed clustered boll-2 (Silow, 1946). *GbAF* was delimited to the region between K2951 and K2834, approximately 0.35 Mb in size (Fig. 1B), using indels based on the TM-1 reference genome (Zhang *et al.*, 2015). This region of the genome contains five annotated open reading frames (ORFs; Fig. 1B).

To identify the candidate causal genes for the *GbAF* phenotype, we assessed 229 ELS accessions, which represented cultivars or lines that had been historically released in China. Of these, 178 displayed the GbAF phenotype and 51 displayed the long-branching phenotype (Supplementary Table S1). The genome of each accession was resequenced with approximately 6.40-fold genome coverage, which generated a total of 3.7 terabases of raw sequence data. These reads were aligned to the TM-1 reference genome sequence (Zhang *et al.*, 2015) for direct comparison as previously described (Fang *et al.*, 2017). A total of 5820045 high-quality SNPs were identified (minor allele frequency >0.05). With a suggestive threshold (*P* < 8.29 × 10^−7^) in a mixed model, a unique signal associated with the GbAF phenotype was identified in D07-15445079 (Fig. 1C). The strongest GbAF phenotype-associated signal was generated by a non-synonymous SNP in Gh_D07G1075, an ortholog of Arabidopsis *TFL1* (Shannon and Meeks-Wagner, 1991; Bradley *et al.*, 1997), *Antirrhinum CEN* (Bradley *et al.*, 1996), and tomato *SP* (Pnueli *et al.*, 1998; Carmel-Goren *et al.*, 2003). We determined the full-length genomic Gh_D07G1075 sequence in WT Hai7124 and found it to be 998 bp in full length, containing a 525-bp ORF consisting of four exons encoding 174 amino acid residues with a predicted molecular mass of 19.69 kDa. Phylogenetic analysis revealed that Gh_D07G1075 is closely related to tomato *SP* (Pnueli *et al.*, 1998; Carmel-Goren *et al.*, 2003) (Supplementary Fig. S3), and thus herein we use *GbSP* for the *G. barbadense* gene and *GoSP* to denote all of *Gossypium* genes as a group. A non-synonymous SNP (from C to T) was identified at the 811-bp position, which resulted in an amino acid substitution from proline (Pro) to serine (Ser) at the 113th position (*Gbsp*^*113Ser*^; Fig. 1D). Gene-based association analysis revealed that all GbAF phenotype accessions, including Xinhai21, TH09-286, Changfeng5, Xinhai10, and Xinhai25, carried this SNP. qRT-PCR analysis showed that *GbSP* temporal–spatial expression in WT Hai7124 was significantly higher than that in the corresponding GbAF-phenotype Xinhai 25 (Fig. 1E). Therefore, *Gbsp*^*113Ser*^ may be a key causal gene responsible for the GbAF phenotype in *G. barbadense*. Furthermore, this non-synonymous SNP was associated with each of the 411 GbAF-phenotype plants in the (TM-1×Xinhai 25)F_2_ segregated population (Supplementary Table S4). Combined, these results indicate the *Gbsp*^*113Ser*^ is a mutant *GoSP* allele responsible for the GbAF phenotype in Central Asian ELS cotton.

### A *GoSP* allele causes the clustered boll phenotype in *G. hirsutum*

**Fig. 2.**
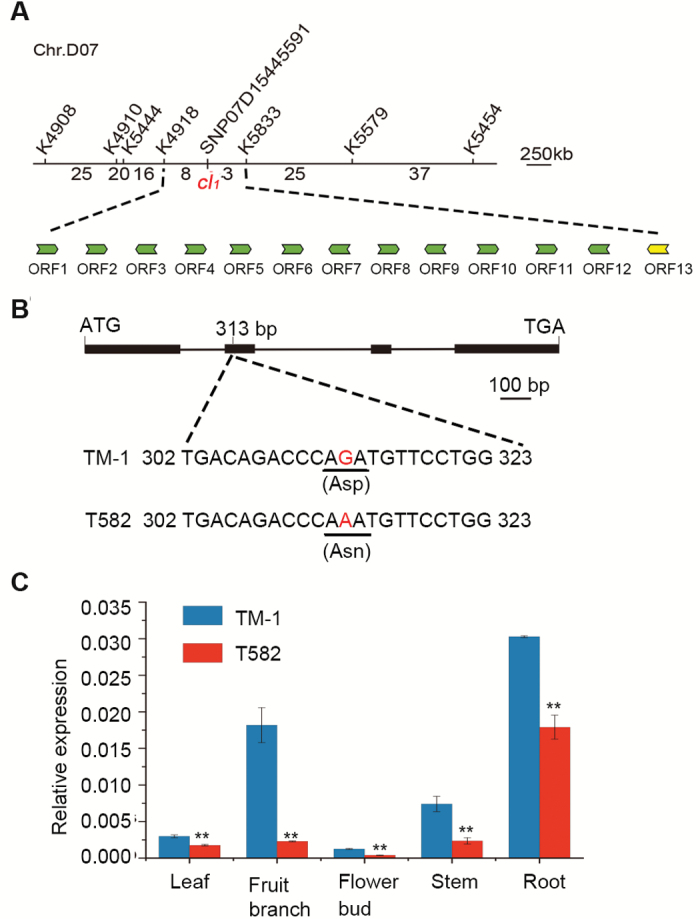
Fine mapping and cloning of the clustered boll-1 mutant allele *cl*_*1*_. (A) Fine mapping of *GhSP*/*cl*_*1*_ with indel and SNP primers. (B) The non-synonymous SNP at position 313 bp of *GhSP*/*cl*_*1*_ led to an amino acid substitution from aspartic acid to asparagine. (C) qRT-PCR analysis of *GhSP*/*cl*_*1*_ gene expression in WT TM-1 and GhCB-phenotype T582 leaf, fruit branch, flower bud, stem, and root tissue. Data are averages of three independent biological replicates ±SEM. Experimental gene expression is normalized to *GhHIS3* (AF024716). **P<0.01 (Student’s *t*-test). (This figure is available in color at *JXB* online.)

The clustered fruiting boll habit typical of the *G. hirsutum* GhCB phenotype, as observed in T582, is caused by monogenic recessive *cl*_*1*_, which is located on chr.D07 and homoeologous to *cl*_*2*_ (Thadani, 1923; Kohel and Lewis, 1984). The GhCB phenotype displays short fruit branches that terminate in a cluster of two or more flowers (Supplementary Fig. S1). Here, *cl*_*1*_ was located to chr.D07 (Hu and Zhou, 2006) (Supplementary Table S4; Supplementary Fig. S4); however, due to the low polymorphism between T582 and TM-1 (Kohel and Lewis, 1984) in the near-isogenic background, it was difficult to fine-map *cl*_*1*_. Therefore, the bulked segregant analysis method was integrated with next generation sequencing techniques to delimit the *cl*_*1*_ locus to between 13.87 and 18.38 Mb on chr.D07 of the TM-1 reference genome (Zhang *et al.*, 2015; Zhu *et al.*, 2017). Within this region, 66 indel markers were further developed (Supplementary Table S2). Using 852 GhCB phenotype plants from segregating (T582×TM-1)F_2_ and BC_1_ populations, *cl*_*1*_ was eventually anchored to an approximate 0.39 Mb interval between the indels K4918 and K5833 (Supplementary Table S4; Fig. 2A). This region contains 13 annotated ORFs (Supplementary Table S6), but only one non-synonymous SNP (from G to A) was identified as being associated with the GhCB phenotype at the 313-bp position (D07_15445591) of Gh_D07G1075, which corresponded to *GhSP* (Zhang *et al.*, 2015).

The deduced amino acid sequences revealed that the non-synonymous SNP caused an amino acid substitution from aspartic acid (Asp) to asparagine (Asn) at position 73 (Fig. 2B). The *GhSP* SNP was identified in 752 GhCB-phenotype plants within the segregating (T582×TM-1)F_2_ and BC_1_ populations (Supplementary Table S4). qRT-PCR analysis showed that the relative expression of *GhSP* in T582 roots, stems, fruiting branches, and leaves was significantly lower than that in TM-1 (Fig. 2C). Therefore, the mutant gene *Gbsp*^*73Asn*^ was considered to be a candidate gene for *cl*_*1*_ associated with the GhCB phenotype. *GhSP* and *cl*_*1*_*/Gbsp*^*73Asn*^ differ only by two base pairs (Supplementary Fig. S5), suggesting that these floral meristem formation genes are well conserved.

A test for allelism revealed that F_1_ plants resulting from a cross between Xinhai 25 (GbAF phenotype) and T582 (GhCB phenotype) all displayed chimeric axillary flowering and/or clustered bolls (Supplementary Fig. S6). Combined, these results indicate that *GbAF*/*Gbsp*^*113Ser*^ and *cl*_*1*_/*Gbsp*^*73Asn*^ are allelic and the various point mutations in these *GoSP* genes are responsible for the GbAF and GhCB phenotypes.

### Silencing of *GoSPs* results in terminal flowers similar to those in GbAF- and GhCB-phenotype plants

**Fig. 3.**
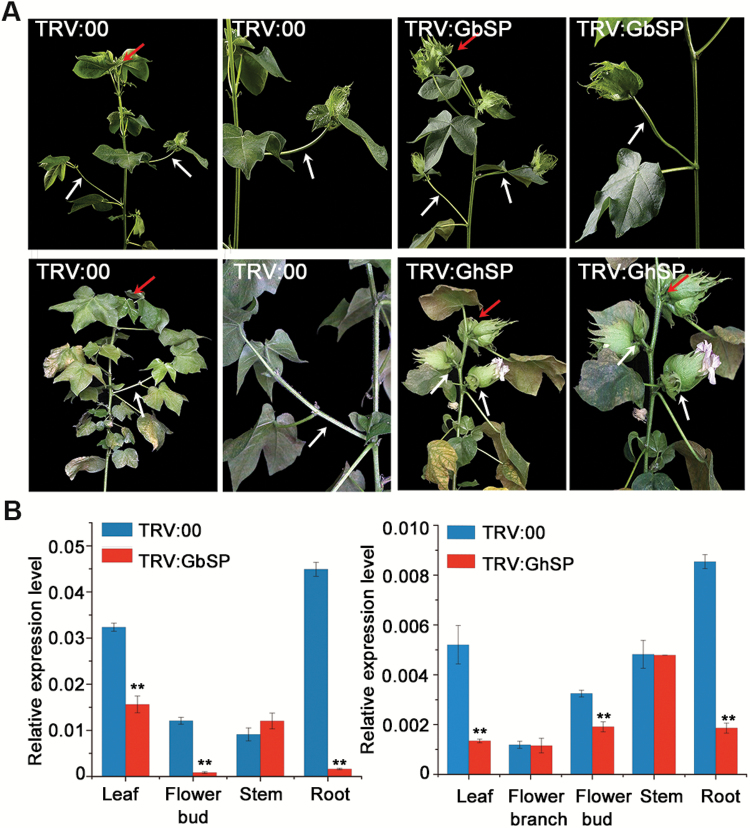
Silencing of *GoSPs* results in terminal flower plants with GbAF and GhCB phenotypes. (A) Phenotypes of *G. barbadense* cv. Hai7124 (the first lane) and *G. hirsutum* acc. TM-1 (the second lane) before and after *GoSP* expression reduction by VIGS, showing the variation in branching patterns. Dark arrow indicates that the apical meristem became terminal flowering in TRV:GbSP or GhSP silencing lines compared with the WT indeterminate growth in the empty vector control TRV:00; white arrow indicates that normal fruit branches became axillary flowering or clustered boll in TRV:GbSP or GhSP compared with TRV:00. (B) Level of *GoSP* transcript in different tissues of *GbSP*/*GhSP*-silenced (TRV:GbSP or GhSP) plants and the negative control (TRV:00). Data are averages of three independent biological replicates ±SEM. Experimental gene expression is normalized to *GhHIS3* (AF024716). **P<0.01, *P<0.05 (Student’s *t*-test). (This figure is available in color at *JXB* online.)

To suppress endogenous *GoSP* expression, 302-bp fragments including the second and the fourth exon of *GoSPs* from WT H7124 and TM-1 were cloned and inserted into pTRV2 for VIGS (Gao *et al.*, 2011). The newly emerging tissue 2 months post-agroinfiltration was then used to assess plant phenotypes. In Hai7124, 45 out of 50 VIGS plants exhibited the GbAF phenotype, whereas 44 out of 50 VIGS plants exhibited the GhCB phenotype in TM-1. All *GoSP*-silenced plants consistently displayed terminal flowers, resulting in a determinate architecture (Fig. 3A). These results are consistent with the functional characterization of *SP* genes as previously reported in tomato (Pnueli *et al.*, 1998) and cotton (McGarry *et al.*, 2016). The phenotypes of these VIGS allotetraploid cottons may be the result of reduced *GoSP* expression from the duplicate loci, comparable to how *SP* mutation in diploid tomato produces a determinate architecture. However, the phenotype of *GoSP*-silenced plants is only moderately similar to that caused by *GbAF*/*Gbsp*^*113Ser*^ and *cl*_*1*_/*Gbsp*^*73Asn*^. Furthermore, species-specific variations were observed between *G. hirsutum* and *G. barbadense*. qRT-PCR analysis revealed that the expression of each respective *GoSP* in leaf, flower bud, and root tissues was much lower in *GoSP*-silenced plants compared with that in Hai7124 and TM-1 (Fig. 3B). However, comparable *GoSP* expression was observed in stem tissues of both *GoSP*-silenced plants and Hai7124. This difference suggests that genetic background may influence *GoSP* gene expression. Our analyses of VIGS plants confirm that *GoSP* is a casual gene for the GbAF and GhCB phenotypes associated with the *Cl*_*1*_ locus. Thus, due to the allelism between *GbAF* and *cl*_*1*_ described above, we renamed *GbAF* as *CLUSTERED BOLL-1*^*Gb*^ (*cl*_*1*_^*b*^).

The development of fruiting branches in New World cotton is controlled by duplicated loci, namely the recessive clustered boll-1 (*cl*_*1*_) on chr.D07 in *G. hirsutum* and clustered boll-2 (*cl*_*2*_) on chr.A07 in *G. barbadense* (Supplementary Fig. S7). A WT allele (*Cl*_*1*_/*Cl*_*2*_) cannot mask a mutant allele (*cl*_*1*_/*cl*_*2*_) at the alternative duplicated locus. qRT-PCR analysis of *GoSP* expression in various *G. hirsutum* tissues at different developmental stages revealed that the *GoSP* gene on chr.A07 was preferentially expressed in stems, leaves, the SAM, and flowers, whereas the *GoSP* gene on chr.D07 was primarily expressed during initial ovule development (Supplementary Fig. S8). These contrasting expression patterns suggest that the duplicated *GoSP* loci have become functionally differentiated in allotetraploid cotton.

### Transgenic expression of *GoSPs* complements the Arabidopsis *tfl1* mutant phenotype and restores indeterminate florescence

To investigate whether *GoSPs* are functional orthologs of Arabidopsis *TFL1* (Shannon and Meeks-Wagner, 1991; Bradley *et al.*, 1997), WT Col-0 and *tfl1* Arabidopsis plants were transformed to overexpress *GoSPs* (Supplementary Figs S9, S10). The *tfl1* mutation causes the conversion of the normally indeterminate inflorescence to a determinate state, which leads to earlier bolting, reduced plant height, and an increased number of rosette inflorescences (Bradley *et al.*, 1997). cDNAs for both *GhSP* and *GbSP*, as well as their corresponding mutant alleles *Ghsp*^*73Asn*^ and *Gbsp*^*113Ser*^, were expressed in the *tfl1-1* mutant under control of the CaMV*35S* promoter. Transgenic constitutional *GhSP* and *GbSP* expression in *tfl1* plants restored an indeterminate florescence phenotype similar to that in WT Col-0, which promoted later flowering with more rosette leaves under long day conditions (Supplementary Fig. S9). However, transgenic *tfl1-1* plants expressing *Ghsp*^*73Asn*^ and *Gbsp*^*113Ser*^ exhibited no obvious phenotypic difference compared with *tfl1-1* (Supplementary Fig. S9A), with similar rosette numbers (Supplementary Fig. S9B) and flowering times (Supplementary Fig. S9C) observed among all plant lines. These results further indicate that *GoSP* is the *TFL1/CEN/SP* homolog.

Constitutive expression of *GoSP* cDNA in WT Col-0 plants led to slightly later flowering with more rosette leaves under long-day conditions compared with that in untransformed plants (Supplementary Fig. S10). However, overexpression of the mutant *Gosps*, *Ghsp*^*73Asn*^ and *Gbsp*^*113Ser*^, could not delay flowering under long day conditions.

### Non-conservative amino acid changes likely change GoSP and FT interactions with FD

A multiple amino acid alignment is presented in Supplementary Fig. S11A, which compares *G. barbadense* GoSPs from the GbAF-phenotype Xinhai25 with those in the WT Hai7124, and *G. hirsutum* GoSPs from the GhCB phenotype T582 with those in the WT TM-1. This analysis revealed two amino-acid sequences (D-P-D-x-P and G-x-H-R) and two key amino-acid sites (His88 and Asp141) that are highly conserved among PEBP family members. His88 in TFL1/SP-like and the corresponding Tyr85 in FT-like is important for the antagonistic interaction between FT and TFL1 (Hanzawa *et al.*, 2005; Ahn *et al.*, 2006). The predicted 3D protein structures of GoSPs revealed a structure that closely resembled that of *Antirrhinum* CEN (Banfield and Brady, 2000; Supplementary Fig. S11B). Gbsp^73Asn^ in T582 and sp^76Leu^ in tomato (Pnueli *et al.*, 1998) are both affected in the conserved D-P-x-P motif, and the mutation within Gbsp^113Ser^ in XH25 is situated close to the G-x-H-R motif. Moreover, Ghsp^73Asn^ and Gbsp^113Ser^ are affected in residues close to the anion binding site in the spatial structure, which may affect GoSP function in cotton.

To analyse whether the mutated SP proteins are affected in their subcellular mechanism and interaction with FD, we created constructs containing GhSP, Ghsp^73Asn^, GbSP, and Gbsp^113Ser^ C-terminal fusions with GFP under control of the CaMV*35S* promoter (Supplementary Table S3). These constructs were transiently expressed in leaf epidermal cells of *N. benthamiana* and subsequent fluorescence signals were monitored by confocal laser scanning microscopy. GFP fluorescence from all SP–GFP fusion proteins was observed in the peripheral cytoplasm (surrounding the vacuole) and in the nucleus, similar to that observed in cells expressing GFP alone (Supplementary Fig. S12), indicating no specific subcellular protein targeting.

Arabidopsis *CENTRORADIALIS* homolog (ATC) interacts with FD and antagonizes FT activity, and also down-regulates the expression of downstream floral meristem identity genes, such as *AP1*, to repress flowering (Huang *et al.*, 2012). Yeast two-hybrid analysis confirmed that both GhSP and GbSP strongly interacted with GhFD, whereas the mutated versions, Ghsp^73Asn^ and Gbsp^113Ser^, displayed no GhFD interaction (Supplementary Fig. S13). These data indicate that Gbsp^73Asn^ in T582 and Gbsp^113Ser^ in Xinhai25 may be affected in their interaction with corresponding FD proteins. Both firefly LCI assays (Chen *et al.*, 2008) and BiFC assays (Waadt *et al.*, 2008) performed in *N. benthamiana* leaf mesophyll cells (Supplementary Table S3) further confirmed these protein interaction results (Supplementary Fig. S14). We observed strong fluorescence in the nucleus of epidermal cells that co-expressed GhSP and GhFD or GbSP and GhFD; however, no fluorescence signal could be detected in epidermal cells expressing Ghsp^73Asn^ and GhFD or Gbsp^113Ser^ and GhFD. These data indicate that neither Ghsp^73Asn^ nor Gbsp^113Ser^ was capable of binding GhFD *in vivo*. Therefore, the presence of Ghsp^73Asn^ or Gbsp^113Ser^ likely promotes a high SFT/FD:SP/FD ratio in meristems, resulting in determinate growth and eventual termination of the apices, causing altered architecture and flower morphology.

## Discussion

### Complex inheritance of plant architecture in tetraploid cottons

In this study, the GbAF phenotype-associated gene *cl*_*1*_^*b*^ and the GhCB phenotype-associated gene *cl*_*1*_ were independently mapped and cloned, and it was found that different allelic variations at the *GoSP*/*Cl*_*1*_ locus were responsible for the different mutant phenotypes. Both cl_1_/Ghsp^73Asn^ in T582 and sp^76Leu^ in tomato (Park *et al.*, 2014) are affected in the conserved D-P-x-P motif (Supplementary Fig. S11A). However, different plant architectures are associated with each mutation. For example, sp^76Leu^ leads to early shoot termination in determinate tomato plants whereas Ghsp^73Asn^ in cotton leads to axillary shoot termination and cluster flowering, while the shoot still displays indeterminate growth. One of the characteristic features of tetraploid cotton is its maintenance of duplicated loci. There are usually two copies of each gene, one each in the A- and D-subgenome chromosomes, representing homoeologous or duplicate copies of the diploid ancestral species’ A and D genomes. Thus, in tetraploid cotton, one monogenic mutation of *GoSPs* may be partly complemented by WT allele at the *Cl* locus in a different subgenome, thus explaining how compact cotton plant architecture with indeterminate shoot growth and determinate flowering habit in fruit branches can arise.

### Natural allelic variation of *GoSP* creates short-branching cotton architecture that facilitates high-density planting for increased yield

Mutation of one homoeologous *SP* in allotetraploid cotton causes a lack of fruit branches and gives rise to cluster bolls in tetraploid cultivars. This compact plant habit greatly improves cotton yield by allowing denser planting, such as is evident in Xinjiang, the currently largest and highest yielding cotton growing area in China. Xinjiang is situated in the north-western short season cotton growing region from N 36°51′ to 46°17′ latitude. In 2016, the cotton unit yield was 1583 kg ha^−1^ in China; however, yields of 1990.9, 1132, and 1088.1 kg ha^−1^ are possible in the Xinjiang Uygur Autonomous Region, the Chinese Yellow River Cotton Growing Region, and the Yangtze River Cotton Growing Region, respectively (http://www.stats.gov.cn), which are all higher than the yield of 899 kg ha^−1^ in the USA (https://www.nass.usda.gov). Because of its high-level production, the cotton growing area in Xinjiang has rapidly increased from 3600 ha in 1950 to 1805200 ha in 2016, and now produces 67.3% of the total cotton yield using only 53.5% the total cotton growing area in China. Notably, cotton yield is proportional to planting density in China. In Xinjiang, the planting density is generally 180000–220000 plants per hectare, which is considerably higher than the 45000–75000 plants per hectare in the Yellow River Cotton Growing Region and the 22000–30000 plants per hectare in the Yangtze River Cotton Growing Region. High planting density contributes greatly towards high cotton fiber yield in Xinjiang. Therefore, a short branch habit represents an ideal plant type for cotton breeding. Almost all the ELS cotton cultivars developed and growing in Xinjiang have a short fruiting-branch habit. Furthermore, many Upland cotton cultivars in this region display a short fruiting-branch habit. The axillary flowering phenotype is the single most important genetic trait concerning cotton production because, in addition to increasing fiber yield, it also potentially facilitates mechanical harvesting. The ideal compact plant type of the GbAF phenotype is being developed in *G. hirsutum* in all cotton growing regions in China, thus allowing denser plantings and mechanical harvesting in these areas. Many short branch cultivars such as Xiangmianzao 1 have already been developed and planted in the Yangtze River Cotton Growing Region and Xinjiang region. Extensive planting of varieties with a short branch habit would represent a novel worldwide cotton growing practice that would increase cotton fiber yield.

### Molecular mechanism of indeterminate inflorescences in cotton

Plant species have two primary types of flowering architecture, namely indeterminate and determinate inflorescences. These two architectures are controlled by the expression patterns of meristem identity genes including *CEN*, *TFL1*, and *SP* (Shannon and Meeks-Wagner, 1991; Bradley *et al.*, 1997; Pnueli *et al.*, 1998; Carmel-Goren *et al.*, 2003). SP-like activities reside primarily in the meristem. FT and TFL1 act as a floral promoter and inhibitor, respectively. A high SFT:SP ratio in meristems promotes determinate growth and eventual termination of the apices, whereas a low SFT:SP ratio promotes indeterminate growth (Shalit *et al.*, 2009; McGarry and Ayre, 2012).

Two motifs, D-P-D-x-P and G-x-H-R, contribute towards conformation of the ligand binding site in CEN (Banfield and Brady, 2000). The mutations within Gbsp^73Asn^ and Gbsp^113Ser^ were spatially close to the anionic ligand-binding site (Supplementary Fig. S11A), suggesting that these mutations may affect the binding of SP proteins and phosphate ions, and thus alter the interaction between GoSPs and GoFD. In Arabidopsis, both TFL1 and ATC compete for interaction with FD, and thus antagonize FT activity in the apex and act as transcriptional repressors of flowering transition, leading to prolonged vegetative growth (Huang *et al.*, 2012). Overexpression of the cotton *FT* orthologue *GhFT* promotes flowering (Guo *et al.*, 2015; Zhang *et al.*, 2016). Furthermore, GhFD was shown to physically interact with GhFT (Zhang *et al.*, 2016). Our study confirmed that GoSPs can interact with GhFD, suggesting that cotton SP homologs share a conserved mechanism for controlling flowering transition.

Neither Ghsp^73Asn^ nor Gbsp^113Ser^ interacted with GhFD *in vivo* (Supplementary Figs S13, S14), suggesting that these mutant proteins cannot compete for interaction with FD and antagonize FT activity to act as transcription repressors of flowering transition, which is consistent with previous results (Pnueli *et al.*, 2001). Mutations in the tomato SP conserved structure domain abolished interactions between SP and its partner proteins, suggesting that CETS in plants have the potential to interact with a variety of signaling proteins. Functional diversification was previously reported between *DETERMINATE* (*DET*) and *LATE FLOWERING* (*LF*), which represent two *CETS* genes in pea (Foucher *et al.*, 2003). Such examples suggest that different species have evolved different strategies to control key developmental transitions. At least eight pairs of CETS homologs exist in allotetraploid cotton (McGarry *et al.*, 2016), and other antagonist molecules in cotton cannot be ruled out.

Tobacco plants overexpressing *GhFT* display lateral outgrowth, leaf morphology alteration, and flower abscission in addition to earlier flowering (Li *et al.*, 2015). Transient transgenic expression of Arabidopsis *FT* in cotton during virus-induced flowering alters plant architecture, reduces indeterminate aerial organs, and disrupts photoperiodic regulation of flowering (McGarry and Ayre, 2012; McGarry *et al.*, 2013). Experiments in Upland cotton using VIGS-based techniques showed that *GhFT* and *GhSP* contribute to differential regulation of monopodial and sympodial growth by controlling apical meristem activities (McGarry *et al.*, 2016). The data presented in the current study demonstrate that cotton orthologs of both *FT* and *SP* control plant branching habit, and can be used to improve cotton plant architecture.

## Supplementary data

Supplementary data are available at *JXB* online.

Fig. S1. The phenotypes of axillary flowering or cluster boll cotton plants.

Fig. S2. Mapping of *GbAF* or *cl*_*1*_^*b*^ gene in *G. barbadense.*

Fig. S3. Phylogenetic analysis of plant CETS (or PEBP) homologs.

Fig. S4. Mapping of *cl*_*1*_ gene *in G. hirsutum.*

Fig. S5. Genome sequence comparison of *GoSP* members.

Fig. S6. F_1_ plants crossed between the *GbAF* Xinhai 25 and the clustered boll T582.

Fig. S7. The genotypes and their genetic relationships among the three mutants and the silenced plants of *GoSP*.

Fig. S8. Expression pattern of *GhSP* homoeologs in different tissues of allotetraploid cotton.

Fig. S9. Overexpressing *GoSPs* complements the Arabidopsis *tfl1* mutant phenotype and restores indeterminacy of the inflorescence.

Fig. S10. Overexpressing *GoSPs* delays flowering in Col-0 Arabidopsis.

Fig. S11. Comparison of the predicted amino acid/3D protein structure of GoSPs.

Fig. S12. Subcellular localization of cotton SP-like proteins.

Fig. S13. Yeast two-hybrid assays.

Fig. S14. Interactions between GoSPs and GhFD *in vivo.*

Table S1. The ELS cultivars and/or lines used in GWAS.

Table S2. Primers used in mapping and cloning of *cl*_*1*_ gene.

Table S3. Primers used for interaction of *GoSPs* with *FD in vivo* and detection of gene expression in wild-type and transgenic plants.

Table S4. Segregation of cluster boll genes in cotton populations.

Table S5. Primers used in mapping and cloning of *GbAF* gene (*cl*_*1*_^*b*^).

Table S6. Candidate region for *cl*_*1*_ mutant loci identified by BSA-seq.

Supplementary Figures and Tables

## Competing financial interests

The authors declare no competing financial interests.

## Author contributions

TZ conceptualized the research program. TZ and XH designed the experiments and coordinated the project. ZS and ZH planted and scored the phenotypes of the 229 ELS cotton cultivars and/or lines in Xinjiang. YH, LF, QW, JC, and BL extracted the high-quality DNA, constructed DNA sequencing libraries, and carried out the genome sequencing and GWAS. ZS conducted mapping and cloning of the *GbAF*/*cl*_*1*_^*b*^ gene. JZ FG, YC, and LC conducted fine-mapping and cloning of the *cl*_*1*_ gene. ZS and JZ performed the virus-induced gene silencing assay. LH and XZ performed Arabidopsis transformation and phenotype analyses, subcellular location, BiFC, LCI and yeast two-hybrid assays. TZ, XH, FZ, HL, and JZ analysed all of the data and wrote the manuscript. All authors discussed the results and commented on the manuscript.

## Abbreviations:

BiFC: bimolecular fluorescence complementation
*cl*_*1*_^*b*^: *clustered boll-1* ^*Gb*^
*cl*_*1*_: clustered boll-1
*cl*_*2*_: clustered boll-2
ELS: extra long staple
GFP: green fluorescent protein
GWAS: genome-wide association study
LCI: luciferase complementation imaging
VIGS: virus-induced gene silencing.
