# Supplementary material for: Mutation of SELF-PRUNING homologs in cotton promotes short-branching plant architecture
Source: J Exp Bot. 2018 Mar 14;69(10):2543–53. doi: 10.1093/jxb/ery093 (PMC5920339; doi:10.1093/jxb/ery093)
Supplement: Supplementary Figures and Tables [file ery093_suppl_supplemental_figures_and_tables.pdf]

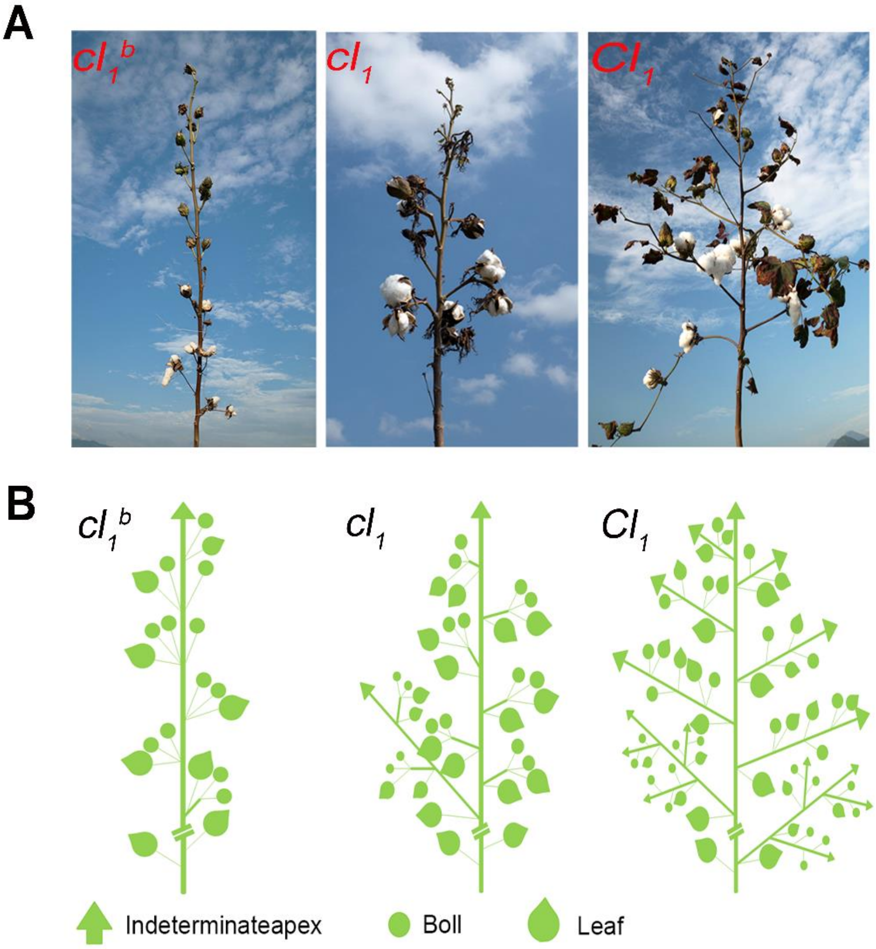

Supplementary Figure S1. The phenotypes of axillary flowering or cluster boll cotton plants. (A)Three branch phenotypes: cl_1^b (left), cl_1 (middle) and Cl_1 (right). (B) Diagram of three branch phenotypes: cl_1^b (left), cl_1 (middle) and Cl_1 (right)

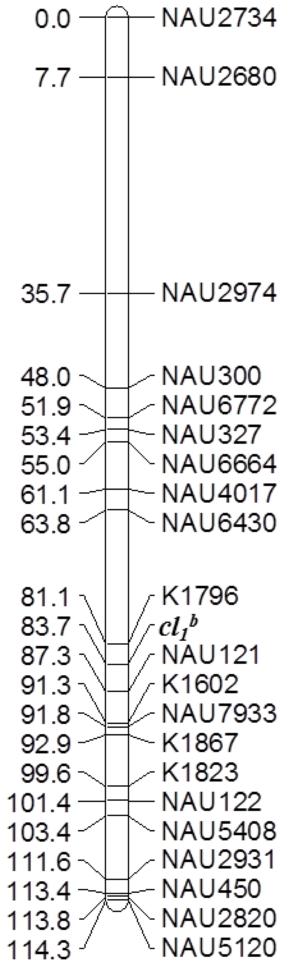

Supplementary Figure S2. Mapping of *GbAF* or cl_1^b gene in *G. barbadense*

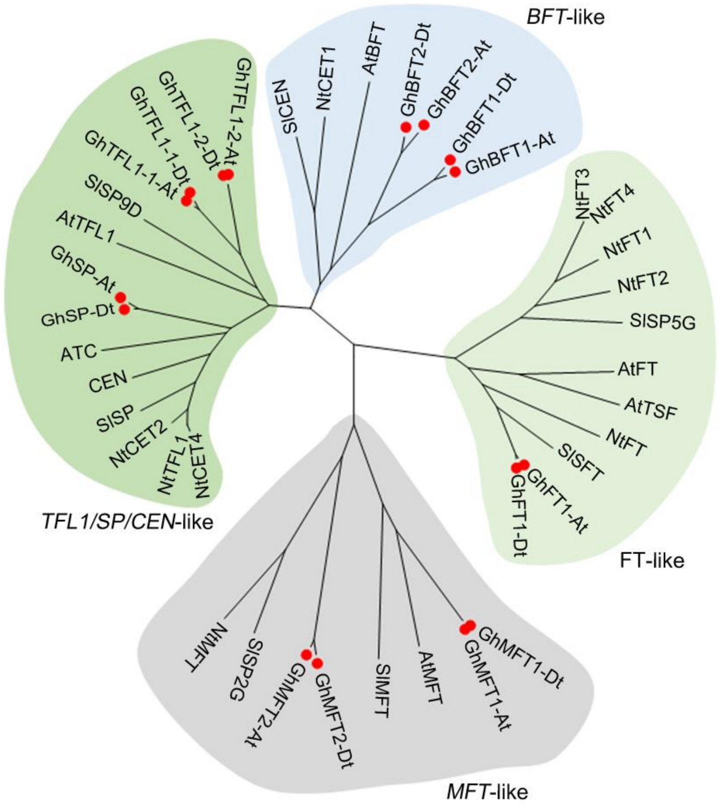

Supplementary Figure S3. Phylogenetic analysis of plant CETS (or PEBP) homologs. The tree was constructed by the Neighbor-Joining (N-J) methods for the deduced amino acid sequence of the *CETS* gene family from cotton (*Gosypium hirsutum*), *Arabidopsis thaliana*, snapdragon (*Antirrhinum majus*), tomato (*Solanum lycopersicum*), and tobacco (*Nicotianatabacum*). Bootstrap values for 1000 re-samplings are shown on each branch. The unit for the scale bar displays branch lengths (0.05 substitutions/site). Red circles represent cotton *CETS* genes.

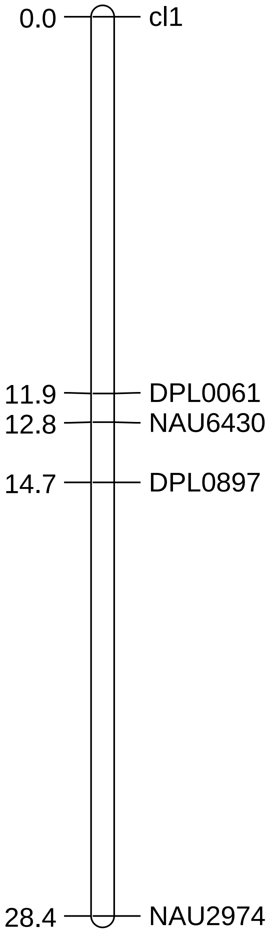

Supplementary Figure S4. Mapping of *cl_1* gene *in G. hirsutum*

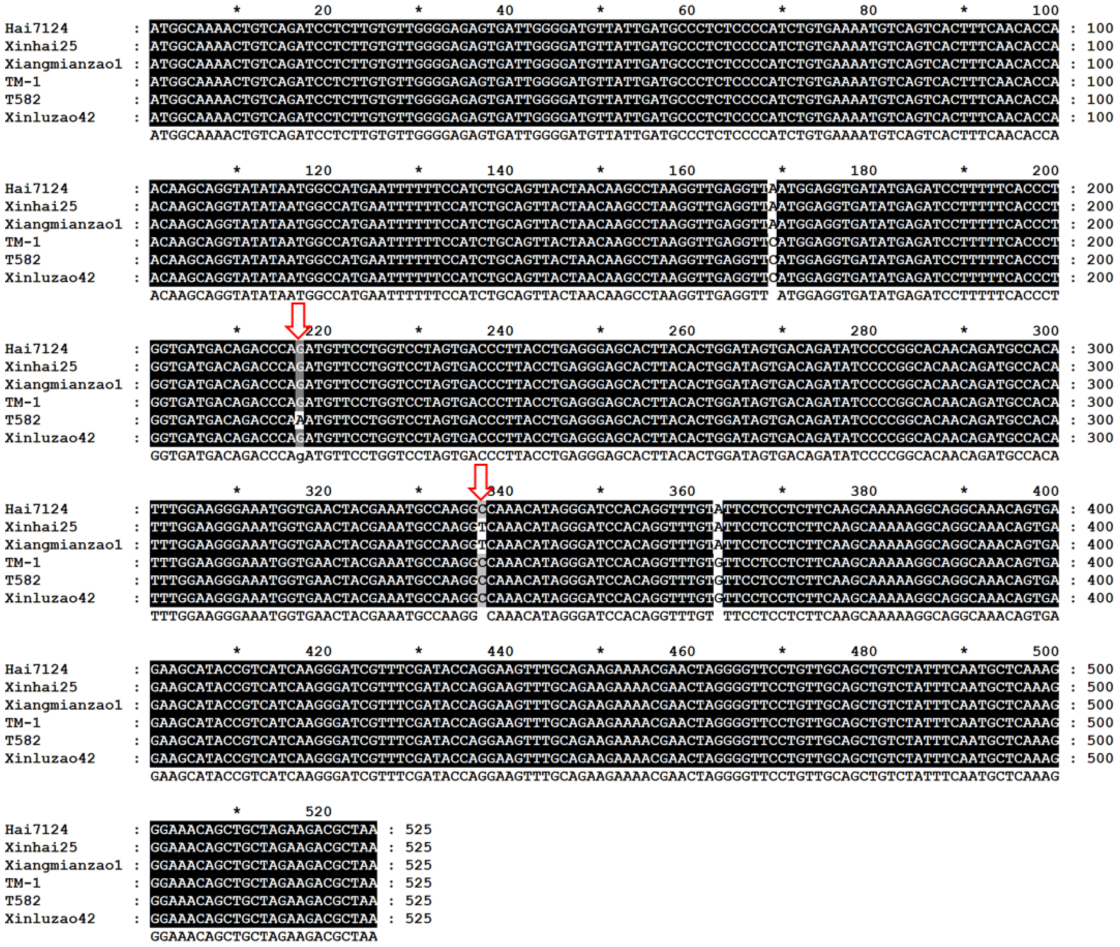

Supplementary Figure S5. Genome sequence comparison of *GoSP* members. The wide type Xianluzao 42 and TM-1, cluster boll T582 and axillary flowering Xianmianzao1 transferred *cl*$_1^b$ from *G. barbadense* by backcrossing are *G. hirsutum* accessions. The axillary flowering Xinhai 25 and the wide type Hai7124 are *G. barbadense* cultivars.

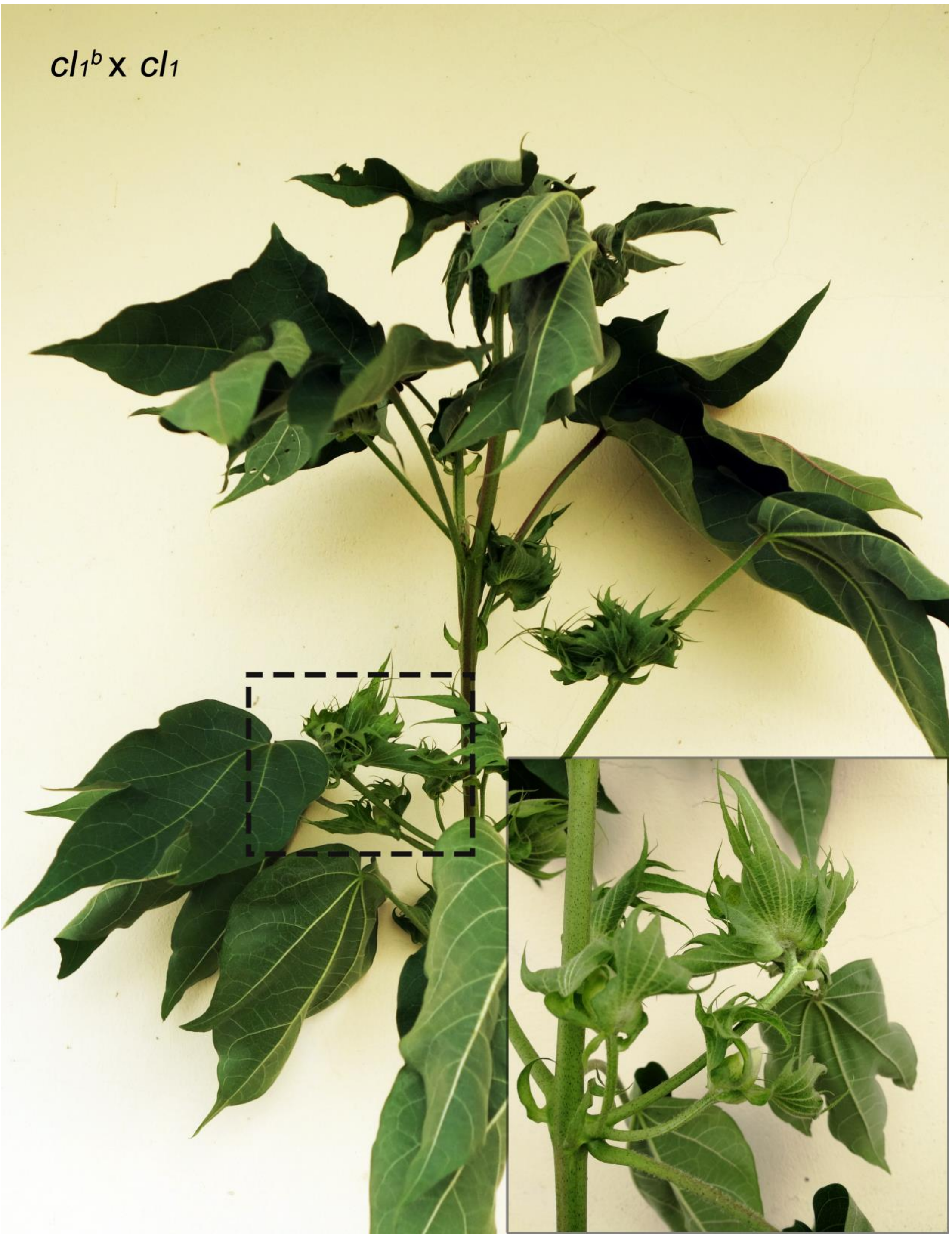

Supplementary Figure S6. F_1 plants crossed between the *GbAF* Xinhai 25 and the clustered boll T582. The box revealed that their F_1 plants were all chimerical axillary flowering and/or clustered bolls.

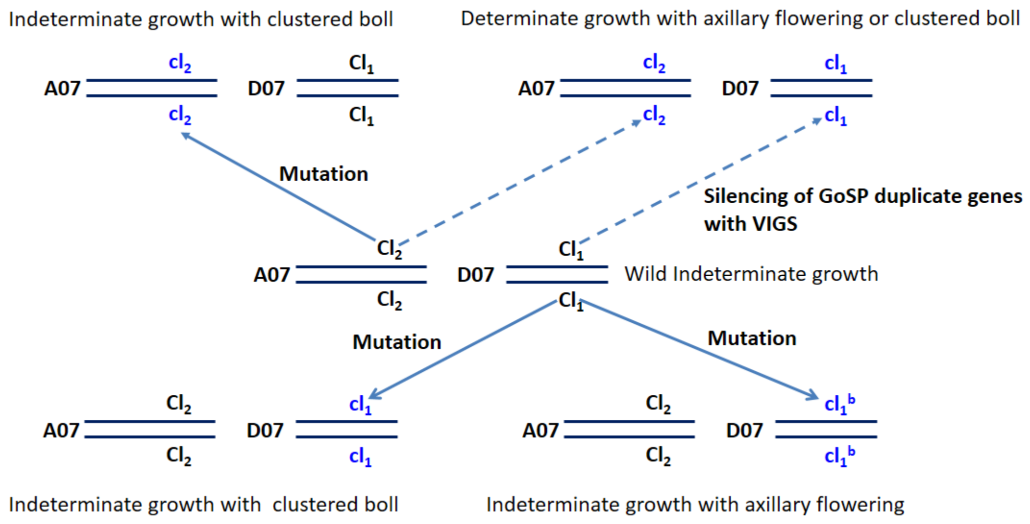

Supplementary Figure S7. The genotypes and their genetic relationships among the three mutants and the silenced plants of *GoSP*. These plants included one with a recessive mutation in At, cl_2Cl_1; two with recessive mutations in Dt, Cl_2cl_1 and $Cl_2cl_1^b$; and one in which *GoSP* duplicate genes were silenced with VIGS, cl_2cl_1.

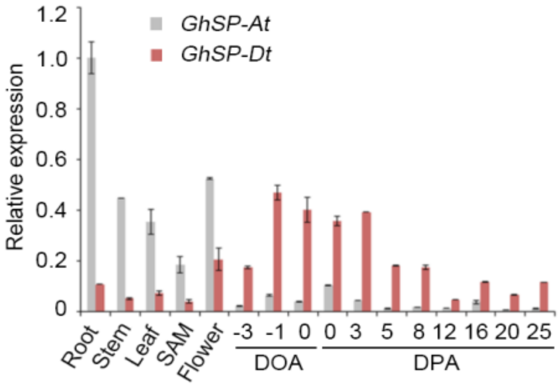

Supplementary Figure S8. Expression pattern of *GhSP* homoeologs in different tissues of allotetraploid cotton. Roots, stems, leaves and shoot apical meristems were sampled at the third true-leaf stage, and a whole flower was collected at the flowering stage. We sampled fiber-bearing ovules on -3, -1, 0 and 1 day of anthesis, 1, 3 and 5 day post-anthesis; 8, 12, 16, 20, and 25 are fiber samples. Data represent the mean ± s.e.m obtained from three independent biological repeats, and *GhUBQ7* (DQ116441) was used as internal control.

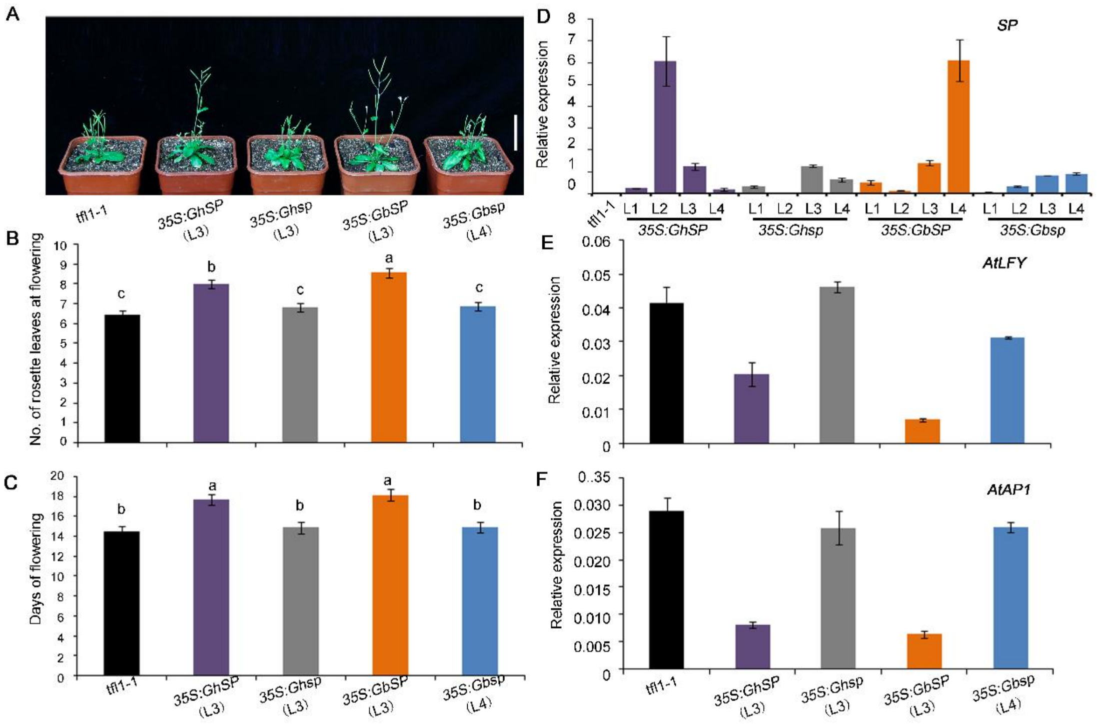

Supplementary Figure S9. Overexpressing *GoSPs*complements the *Arabidopsis tfl1* mutant phenotype and restores indeterminacy of the florescence. (A) Phenotypes of the *tfl1-1* mutant plants and transgenic *tfl1* plants expressing *35S:GoSP* vectors under long-day conditions. Shown are *Arabidopsis* 24-day-old plants grown in a growth chamber under long-day conditions. Scale bar, 4 cm. (B) Flowering time in representative lines as determined by number of rosette leaves at flowering. (C) The days to flowering as determined by the day floral buds became visible. All data presented in B and C are averages from independently transformed lines each containing 12Km-resistant T2 plants for four transgenic events expressing *35S:GoSPs* using the*tfl1* mutant as receptor (lines ± SEM). Different lowercase letters represent statistically significant differences as determined by one-way ANOVA ($P< 0.05$, Duncan's multiple range test). qRT-PCR analysis to confirm the T1 transgenic lines (D); qRT-PCR analysis *AtLFY* and *AtAP1* expression levle in different transgenic *Arabidopsis* lines. In this analysis, *Atactin-2* was used as a reference transcript (E, F).

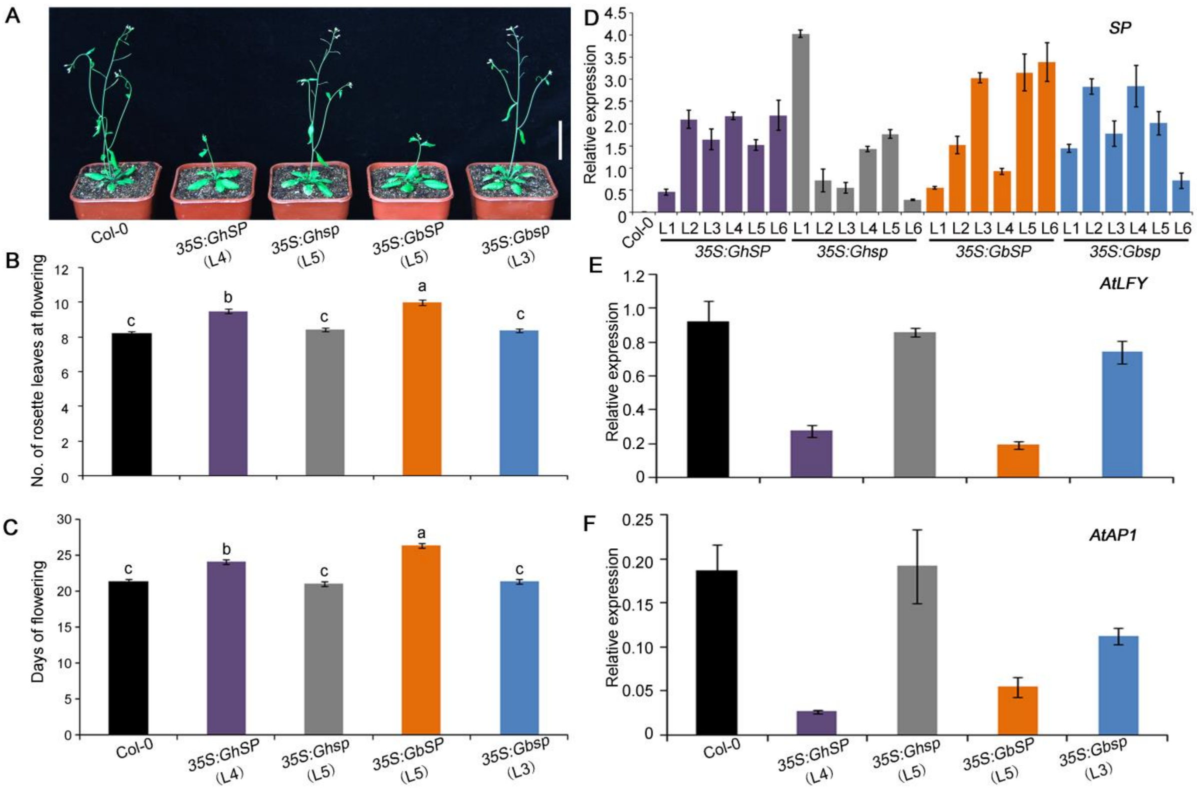

Supplementary Figure S10. Overexpressing *GoSPs* delays flowering in Col-0 *Arabidopsis*. (A) Phenotypes of the wild-type (Col-0) and transgenic *Arabidops* plants expressing *GoSPs* and *Gosps*. Shown are *Arabidopsis* 28-day-old plants grown in a growth chamber under long-day conditions. Scale bar, 4 cm. (B) Flowering time in representative lines as determined by number of rosette leaves at flowering. (C) The days to flowering as determined by the day floral buds became visible. qRT-PCR analysis to confirm the T1 transgenic lines (D); qRT-PCR analysis *AtLFY* and *AtAP1* expression levle in different transgenic *Arabidopsis* lines. In this analysis, *Atactin-2* was used as a reference transcript (E, F). All data presented in B and C are averages from eight independently transformed lines each containing 12 T2 Km-resistant plants for four transgenic events overexpressing *35S:GoSPs* using the Col-0 as receptor (lines ± SEM). Different lowercase letters represent statistically significant differences as determined by one-way ANOVA (*P*< 0.05, Duncan's multiple range tests).

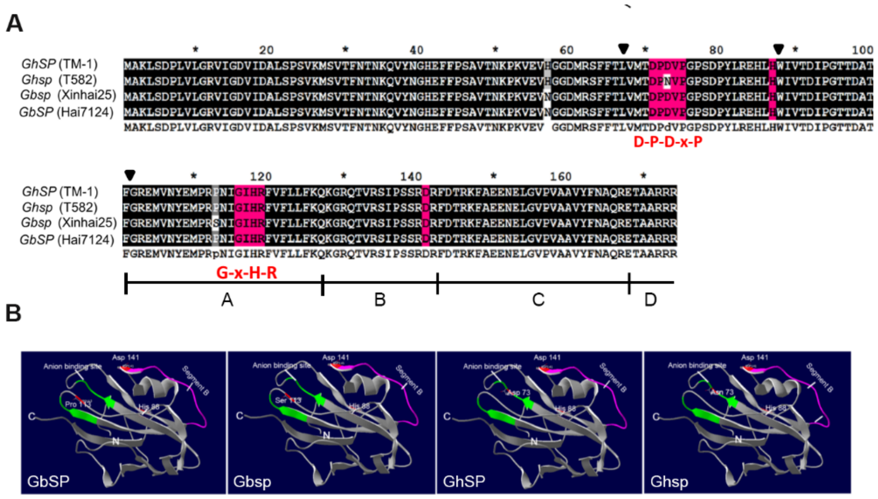

Supplementary Figure S11. Comparison of the predicted amino acid/3D protein structure of GoSPs. (A) The region denoted in red present two conservative motif: D-P-D-x-D and G-x-H-R; the symbol ▼distinguishes the four exons of the genes. A to D denote the four sections of the fourth exon. (B) Comparison of the 3D protein structure of GoSPs, showing that the histidine and aspartic acid residues are located in the vicinity of the pocket. Segment B is colored pink, D-P-D-x-P and G-x-H-R conservations motif are colored green. Amino (N) and carboxy (C) termini are labeled.

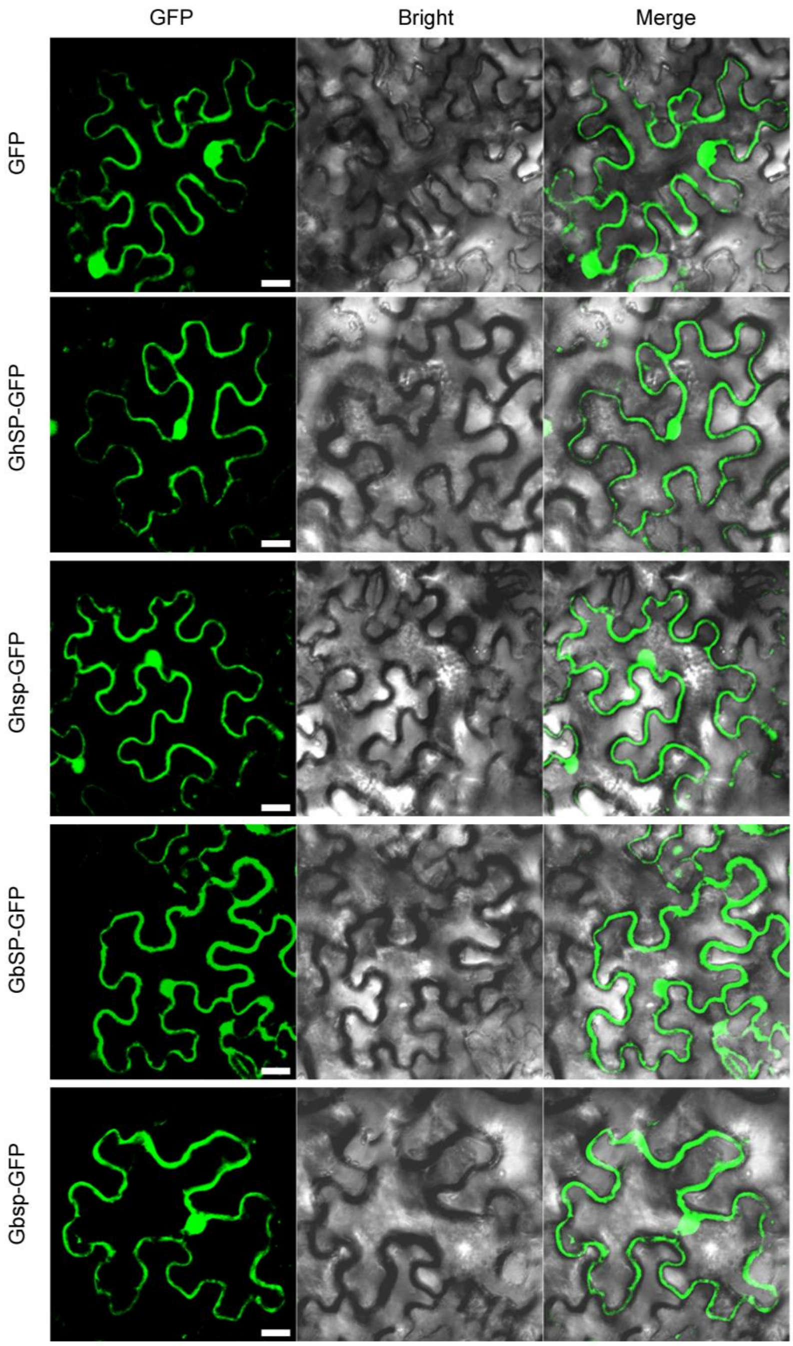

Supplementary Figure S12. Subcellular localization of cotton SP-like proteins. Subcellular localization of GFP, GhSP-GFP, Ghsp-GFP, GbSP-GFP, and Gbsp-GFP of *Nicotiana benthamiana* leaf epidermal cells in which *35S:GFP* and *35S:GoSPs-GFP* expressed. Scale bar, 20 μm.

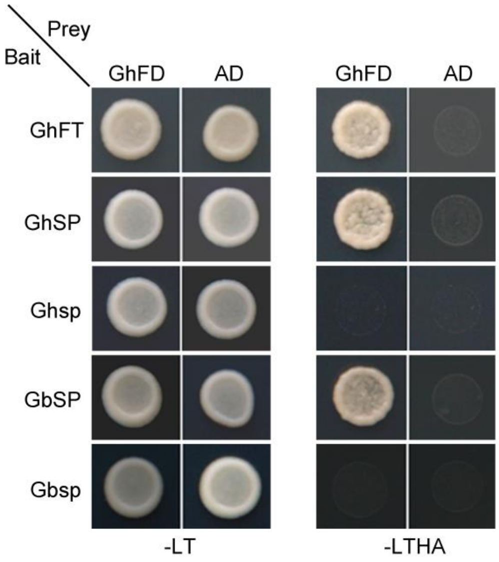

Supplementary Figure S13. Yeast two-hybrid assays. Yeast two-hybrid assay of the interaction between GhFT, GoSPs and GhFD, respectively. Transformed yeast cells harboring GhFD protein fused to AD (activation domain), and GhFT, GoSPs fused to BD (binding domain) were grown on selective media without Leu, Trp, His and Ade (-LTHA) indicating positive interactins.

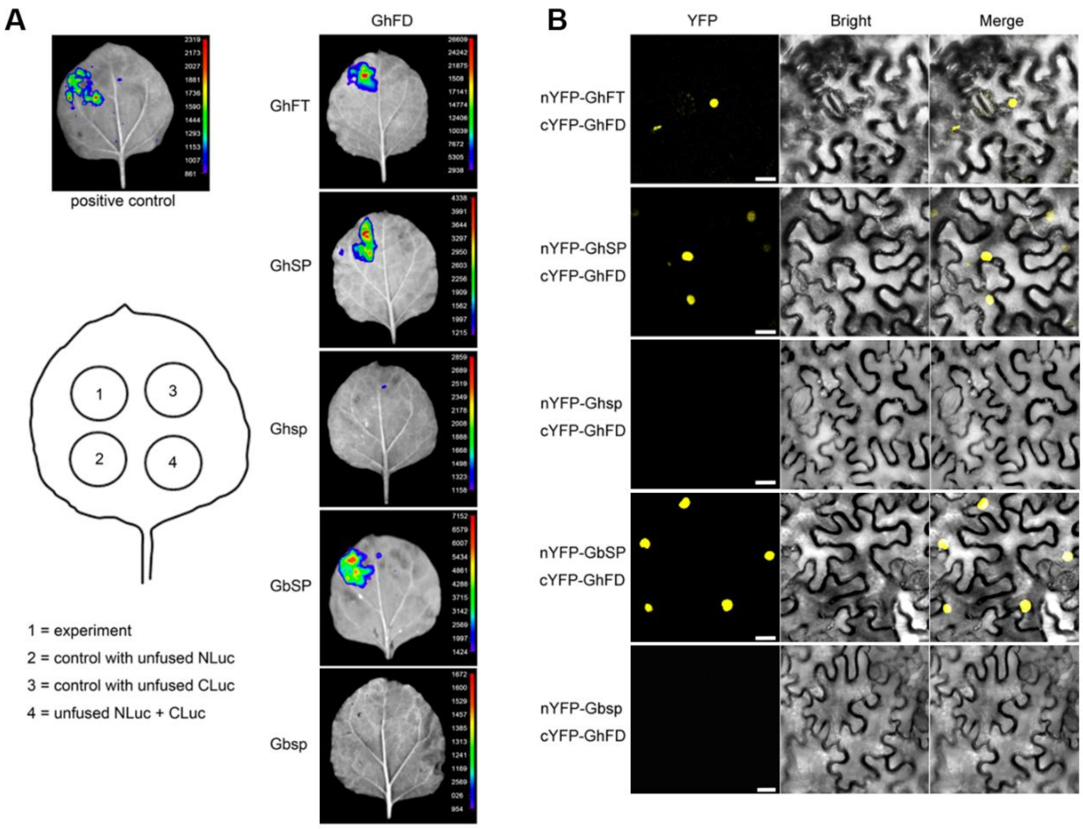

Supplementary Figure S14. Interactions between GoSPs and GhFD *in vivo*. (A) Bimolecular luciferase complementation assays. LUC image of *N. benthamiana* leaves co-infiltrated with Agrobacterium strains containing GoSPs-NLuc and CLuc-GhFD vectors. The color bar on the right shows the range of luminescence intensity in each image (counts per second). (B) BiFC analysis of interactions between GhFD and GhFT with GhSP, Ghsp^{73Asn}, GbSP, and Gbsp^{113Ser} in *N. benthamiana* leaf epidermal cells. Scale bar, 20 μm. Experiments were repeated twice.

Supplementary Table S1. The ELS cultivars and/or lines used in GWAS

Number	Cultivars Name	Phenotype of branches
A001	Jizha45	1
A002	Bahtani	0
A003	Sudanmian	1
A004	Xinong27	0
A005	TH09-286	0
A006	Changfeng3	0
A007	Yuanlong12	0
A008	S10-132	0
A009	Yuanmou1	0
A010	Changrong1	0
A011	Xibeihaidaomian	1
A012	A6303	0
A013	Minufei	0
A014	Changfeng5	0
A015	Pima3	0
A016	Luoxiya1	0
A017	02NH-10	0
A018	08NH-3	0
A019	Changrong12	0
A020	07NH-17	0
A021	08NH-7	0
A022	AW2013	0
A023	H33790	0
A024	Lutai572Q	0
A025	07NH-68	0
A026	06NC-12	0
A027	07NH-15	0
A028	08NH-16	0
A029	07NH-20	0
A030	HS11-3	0
A031	Y14-10	0
A032	Ta09-63	0
A033	Yuanlong5	0

A034	k-412	0
A035	XLD66	0
A036	Jiumian85	0
A037	Xinhai41	0
A038	H33781	0
A039	DJ08-378	0
A040	DJ08-378	0
A041	JSH12-3	0
A042	P3-79	0
A043	Y14-3	0
A044	XLD58	0
A045	G-92	0
A046	XLD20	1
A047	XLD14	1
A048	8-11H	0
A049	XLD48	1
A050	ChangA11-3	0
A051	08NH-2	0
A052	07NH-13	0
A053	XLD17	1
A054	XLD4	1
A055	XLD18	1
A056	Xinhai26	0
A057	XLD40	1
A058	XLD15	1
A059	Xinhai28	0
A060	XLD45	1
A061	XLD56	0
A062	Xinhai15	0
A063	Changfeng1	0
A064	07NH-5	0
A065	Yuanlong11	0
A066	16-9H	0
A067	Qianjin616-3-2	0
A068	2-9H	0

A069	XLD7	1
A070	XLD6	0
A071	Xinhai20	0
A072	XLD13	1
A073	XLD3	1
A074	XLD35	1
A075	XLD29	1
A076	Xinhai44	0
A077	XLD44	0
A078	XLD53	0
A079	XLD34	1
A080	25-6H	0
A081	XLD31	1
A082	Xinhai34	0
A083	XLD55	0
A084	XLD38	1
A085	07NH-16	0
A086	AW-2044	0
A087	Xinhai40-1	0
A088	Xinhai39	0
A089	Xinhai40-2	0
A090	DJ09-172	0
A091	Yuanlong10	0
A092	Jinken1354	0
A093	Ta09-164	0
A094	3-2H	0
A095	TH-314	0
A096	Changfeng2	0
A097	Changfeng4	0
A098	Xinhai42	0
A099	02NH-20	0
A100	H3549	0
A101	Xinhai6	0
A102	Y14-25	0
A103	k-138	0

A104	Yuanlong13	0
A105	Y14-9	0
A106	08NC-25H	0
A107	TH08-285	0
A108	DJ9031	0
A109	Xinhai8	0
A110	Y14-28	0
A111	Y14-27	0
A112	Y14-18	0
A113	04NC-24H	0
A114	98107	0
A115	Jizha81	1
A116	Pima	1
A117	Pima5	1
A118	08NH-42	0
A119	HS12-5	0
A120	9108	0
A121	04NC-20H	0
A122	07NC-33H	0
A123	Ta09-324	0
A124	Ta10-280	0
A125	08H-6	0
A126	Xinhai35	0
A127	33687	0
A128	K399	0
A129	06NH-2	0
A130	Changrong4	0
A131	Y14-1	0
A132	XLD8	1
A133	Y14-4	0
A134	XLD12	1
A135	Y14-13	0
A136	Y14-7	0
A137	Y14-21	0
A138	X-2038	0

A139	XLD28	1
A140	7-4H	0
A141	Tianchang10	0
A142	09NH-44	0
A143	03H-1	0
A144	XLD10	1
A145	XLD39	1
A146	Tianchang16	0
A147	XLD11	1
A148	XLD49	1
A149	XLD27	1
A150	XLD42	1
A151	Ba202	0
A152	XLD59	0
A153	03NH-7	0
A154	P90	0
A155	XLD57	0
A156	Xinhai14	0
A157	Y14-14	0
A158	Y14-24	0
A159	Y14-26	0
A160	Y14-15	0
A161	Xinhai22	0
A162	Y14-16	0
A163	H858	0
A164	Y14-11	0
A165	SDH1161	0
A166	XLD19	1
A167	Y14-2	0
A168	XLD46	0
A169	Y14-8	0
A170	Xinhai32	0
A171	S0717	0
A172	Y14-6	0
A173	Y-163	0

A174	XLD26	1
A175	XLD23	1
A176	XLD45-2	1
A177	167	0
A178	XLD5	0
A179	XLD21	1
A180	XLD52	0
A181	07N-89H	0
A182	07NH-59	0
A183	Xinhai28	0
A184	07NH-59-2	0
A185	118	0
A186	XLD51	0
A187	XLD24	1
A188	XLD37	1
A189	H8645	0
A190	06NH-16	1
A191	B3029	0
A192	H12-640	0
A193	MC-203	0
A194	XLD43	0
A195	Y14-22	0
A196	DJ10149	0
A197	11-616	0
A198	H5161	0
A199	Yuejin2	0
A200	Jizha82	1
A201	uoc620	1
A202	Pima4	1
A203	Jizha36	1
A204	Daxuan71	1
A205	Pima2	0
A206	Jizha67	0
A207	Xinhai4	0
A208	Yue51-11	1

A209	SovietB51(91-438）	0
A210	5320-fu	0
A211	Xinhai27	0
A212	Xinhai24	0
A213	Achang599	0
A214	SovietB69(91-470)	0
A215	Xinhai12	0
A216	Xinhai31	0
A217	Tianchang2	0
A218	DJ-1	0
A219	K366	0
A220	Xinhai18	0
A221	Jiangsuchangrongmian	0
A222	Pima1	1
A223	Jizha80	1
A224	J917	0
A225	Ta07-152	0
A226	H7124	1
A227	Jizha29	1
A228	Xinhai17	0
A229	14XJ	1

Note: "0" stands for monopodial branch and "1" stands for normal branch

Supplementary Table S2. Primers used in mapping and cloning of *cl_1* gene

Purpose	Primer names	Sequence(5'-3')
Indel primers for mapping cl_1	K4908F	TGTTGTTTTTACCTCCATCCAAAGT
Indel primers for mapping cl_1	K4908R	GGCTGGTTTGGTTAATACCCG
Indel primers for mapping cl_1	K4909F	TGACGGATTTGTGATCATCTGT
Indel primers for mapping cl_1	K4909R	CTCAGGCTTAGCTGATCCGG
Indel primers for mapping cl_1	K4910F	CCCCCAAAATAGGTAAAGAGGT
Indel primers for mapping cl_1	K4910R	ACTTCCTCACTTGGCGTCAA
Indel primers for mapping cl_1	K4911F	TCAAATTTTCATGTTGTGCTCGA
Indel primers for mapping cl_1	K4911R	AGTTTGGGAGTGTAATGTGCCT
Indel primers for mapping cl_1	K4912F	AGCCTGCTTTTTAGGTTTGGC
Indel primers for mapping cl_1	K4912R	GGTATTTGCTTGCTAAGTTGGACT
Indel primers for mapping cl_1	K4913F	CGCCGCAAATACCTTTAGCG
Indel primers for mapping cl_1	K4913R	CACCGGTGTAATGGATGCCT
Indel primers for mapping cl_1	K4914F	TGTCATATTCATGTTGGTTTTCGCA
Indel primers for mapping cl_1	K4914R	TCTTACCGATCTCTTCCAAAAATCA
Indel primers for mapping cl_1	K4915F	GCACCGAGATGAGAACTCCC
Indel primers for mapping cl_1	K4915R	ACTTACTCCTCCATTGCTGAAA
Indel primers for mapping cl1	K4916F	TGTCATGAGTTGTGATTAGGGTCA
Indel primers for mapping cl_1	K4916R	TGCGACCAAACATGTAAGTTCA
Indel primers for mapping cl1	K4917F	AAGGGGAACAGATTCGGCTG
Indel primers for mapping cl1	K4917R	CCAAACAGTCCCTAACCCCC
Indel primers for mapping cl1	K4918F	CCGACAATGTGGAAGGAAGC
Indel primers for mapping cl1	K4918R	GCAACTCTCAACAACCAACCT
Indel primers for mapping cl1	K4919F	TTAGGGAAAGCCAGCTCAGC
Indel primers for mapping cl1	K4919R	TGGTTCTGGGGTCTGTAAGT
Indel primers for mapping cl1	K4920F	AGAGTTGTGTGACCCAAATTCT
Indel primers for mapping cl1	K4920R	CACGGGCAGAGGAGAAGAAA
Indel primers for mapping cl1	K4921F	TTAGGAGGGGGAATTCAAATGT
Indel primers for mapping cl1	K4921R	ACACTTTACCCCTTCCCAAAA
Indel primers for mapping cl1	K4922F	ACAAATAACACAAGTTGAAAGGCT
Indel primers for mapping cl1	K4922R	GGTTTAAGACTTCAAAAGAGTAGGGT
Indel primers for mapping cl1	K4923F	ACATCTTTACAATTGTTTCGAGCA
Indel primers for mapping cl1	K4923R	TCCCTCTGAACACATGTTTCGT
Indel primers for mapping cl1	K4924F	TCACCTGCAAGTCTCCGAAG
Indel primers for mapping cl1	K4924R	GGGGTGCATGGTGGACATTA
Indel primers for mapping cl1	K4925F	AGGGTGAGCATTCGATCGAA
Indel primers for mapping cl1	K4925R	CGATGGGTGACAGTGGTTACA
Indel primers for mapping cl1	K4926F	GCCACAGACACAGACACAGA
Indel primers for mapping cl1	K4926R	ACACACACACAAACACACACA
Indel primers for mapping cl1	K4927F	AGCTGTCGGATTGTGATTCCA
Indel primers for mapping cl1	K4927R	TCTTCAAAAACTATCCCAACCCA
Indel primers for mapping cl1	K4928F	ATGGGAGCCTCTCTGGACAT
Indel primers for mapping cl1	K4928R	TCATACCAACATCCCGGGAC

Indel primers for mapping cl1	K4929F	GTCTGTAGCAGCGTCACTCA
Indel primers for mapping cl1	K4929R	ACACACTTGTTGCTTTCAGCC
Indel primers for mapping cl1	K4930F	GCATCCTGCTTTACTTTGTTCAGA
Indel primers for mapping cl1	K4930R	TTTTTGCCTGGCCCATGAAC
Indel primers for mapping cl1	K4931F	GGGGCATTGTCCTTTTGTGG
Indel primers for mapping cl1	K4931R	AGGTCTAGGCTCCTTAGTGGG
Indel primers for mapping cl1	K5444F	TCAGCTTTTCCACCTTGTTCA
Indel primers for mapping cl1	K5444R	AAGCCCCAATGTGAGAGCAA
Indel primers for mapping cl1	K5445F	AGGGAGAAGGCCTCAAATCT
Indel primers for mapping cl1	K5445R	GGAGATGTCTCTGGTCTTGCT
Indel primers for mapping cl1	K5446F	GGGGTCTTTCTGTCCTCAGC
Indel primers for mapping cl1	K5446R	CTGCACATACCCGGTTGACT
Indel primers for mapping cl1	K5447F	TGGTGTATTCATATGCGCAAACT
Indel primers for mapping cl1	K5447R	ACACGCTCAATACAAATCAACTT
Indel primers for mapping cl1	K5448F	TCGATTGTATTTTGGGGGTGA
Indel primers for mapping cl1	K5448R	TTCGTCGGTAAGTCCCCTCT
Indel primers for mapping cl1	K5449F	AGAAAGCTTTAAAGTTGAAGGACTAA
Indel primers for mapping cl1	K5449R	ACTTCCACGCGGTTATGTGA
Indel primers for mapping cl1	K5450F	AGCCCAATACGTTAGGGCAC
Indel primers for mapping cl1	K5450R	TCTTCGAGAGGATTGAGCCC
Indel primers for mapping cl1	K5451F	TCTACTCAGAGTGGACAGCCA
Indel primers for mapping cl1	K5451R	TCCATCCATGCCATTGCTGT
Indel primers for mapping cl1	K5452F	TCCTCGTTACTCTCAGCAGC
Indel primers for mapping cl1	K5452R	GCAAAGCAGATAGGCAAGCT
Indel primers for mapping cl1	K5453F	CCCGAAAAACAAAGAACCCTCA
Indel primers for mapping cl1	K5453R	TCTGTTAGAGTTGTGTGACCCA
Indel primers for mapping cl1	K5454F	TATTTGTATTGGCCCGGGCA
Indel primers for mapping cl1	K5454R	ATGATCCCCGAGCTTGAAGC
Indel primers for mapping cl1	K5455F	TGTTTTAGGGGCTAAAATTGAGGA
Indel primers for mapping cl1	K5455R	TGATGCTGATGCCGGTTCTT
Indel primers for mapping cl1	K5456F	TTCAAATCCCGGCCATTCCA
Indel primers for mapping cl1	K5456R	ACTGTAAATTGAGGGAAAAACAGTGA
Indel primers for mapping cl1	K5573F	GATCCAGTCGAGCGTGTCAA
Indel primers for mapping cl1	K5573R	GCATTTGAGGCCATGACTCG
Indel primers for mapping cl1	K5574F	CGGCAGATTCTAGGTTCCGA
Indel primers for mapping cl1	K5574R	TTCCGAGGTCCCAAAACACT
Indel primers for mapping cl1	K5575F	TTCACGATGAAACCAAAATCTTCA
Indel primers for mapping cl1	K5575R	TCGTTGATCAAGCTGTTTTTGTGA
Indel primers for mapping cl1	K5576F	TGTTTGGCCTTGGCATTTCG
Indel primers for mapping cl1	K5576R	TATCCCCGGCACAACAGATG
Indel primers for mapping cl1	K5577F	TGGAAACAGAGGAAGTGGCA
Indel primers for mapping cl1	K5577R	GTCAGCCCGAGAGCATTGT
Indel primers for mapping cl1	K5578F	GTAGCCCGATGTGCTCTTCA
Indel primers for mapping cl1	K5578R	ACCTGACAACAACAAGGCCA

Indel primers for mapping cl1	K5579F	TGTCTTTGGGCTTTTGAACTTGT
Indel primers for mapping cl1	K5579R	CAAATTATAATTTTGCTCGGCACGT
Indel primers for mapping cl1	K5580F	ATCAGCCAAGTTCTCCGCAA
Indel primers for mapping cl1	K5580R	CACCTCCGCTTCAATGGGAT
Indel primers for mapping cl1	K5581F	TCCGCGTTTGGGTATTTTGC
Indel primers for mapping cl1	K5581R	ACCCCCTACAATTTCCAACCA
Indel primers for mapping cl1	K5582F	CCGGCAACTAGTCTGCTAGG
Indel primers for mapping cl1	K5582R	TGATTGAACATGGACTACAACGA
Indel primers for mapping cl1	K5583F	GCCGCCGTCTTAGACTATCG
Indel primers for mapping cl1	K5583R	CACCTTTAGTGGCGATTTGGC
Indel primers for mapping cl1	K5584F	AGTTCAACCGGGAATTTACACT
Indel primers for mapping cl1	K5584R	GGAATCGACCTTGACCGGAG
Indel primers for mapping cl1	K5826F	TGACGACGGCTCTTAACAGG
Indel primers for mapping cl1	K5826R	GCCCCAAACACCTGCAAAAA
Indel primers for mapping cl1	K5827F	TGTGGACGTCGACACTTCAG
Indel primers for mapping cl1	K5827R	CTCTAGTCGGTGCTCATGCC
Indel primers for mapping cl1	K5828F	CCGTAAGTCATTCTTCGATTCAGT
Indel primers for mapping cl1	K5828R	TGTGTTGATCAGATCTCCCGA
Indel primers for mapping cl1	K5829F	AGCAAGCCAATGGTTCCAAA
Indel primers for mapping cl1	K5829R	TCTGTTAATGCCACTATGCAGT
Indel primers for mapping cl1	K5830F	CCGAAACATGTCAAACAGAAGCT
Indel primers for mapping cl1	K5830R	TGGCTGTTTCGGTTGGATCA
Indel primers for mapping cl1	K5831F	TGCTAACAGTTTCCCCGGAC
Indel primers for mapping cl1	K5831R	TGCACAACGGGATTATGTGA
Indel primers for mapping cl1	K5832F	CTAAGCCGGACCTAGCCTTG
Indel primers for mapping cl1	K5832R	AGAGACTAGTGGTAGCACTGT
Indel primers for mapping cl1	K5833F	CGCGACGACTTAGGTACGTT
Indel primers for mapping cl1	K5833R	GCCGATCCCTGCAAACAAAT
Indel primers for mapping cl1	K5834F	ACACACTTCCAAAATAGGTCTAGA
Indel primers for mapping cl1	K5834R	AAGTGAACTCGGTTATGGCA
Indel primers for mapping cl1	K5835F	TGACATTCCATCCTTGAGTCATCA
Indel primers for mapping cl1	K5835R	AGTGAGAAAAGTTTTGTTGCCCA
Indel primers for mapping cl1	K5836F	TGGTTAGCTTCTTTGCTTGCT
Indel primers for mapping cl1	K5836R	ATTCCCTTCCCATCCCACCT
Indel primers for mapping cl1	K5837F	GTCGGCAAGCCACTTGAAAA
Indel primers for mapping cl1	K5837R	GGCAGACAGTTGAGTCCGAA
Indel primers for mapping cl1	K5838F	AACAACGAGCTGCCTATGCT
Indel primers for mapping cl1	K5838R	TTGGTTTCCTGCATGGTGGA
Indel primers for mapping cl1	K5839F	CGAGCCCTAACGTATTGGGT
Indel primers for mapping cl1	K5839R	ACATGTCACATCCAATGCGT
Indel primers for mapping cl1	K5840F	TCAGGATGGTGGAAATCATGTCT
Indel primers for mapping cl1	K5840R	AAGCCTCCCATTGCCTTCAA
Indel primers for mapping cl1	K5841F	AGTGTCAAGTCCAAGCTGCA
Indel primers for mapping cl1	K5841R	ATGGTGTCAGCTGTGCAGAT

Indel primers for mapping cl1	K5842F	AGGACAAGAGCGCCATTACC
Indel primers for mapping cl1	K5842R	AGGCATTGGTCAACTAAGGTGT
SNP primers for mapping cl1	SNPD07_15445591F	TCAGGTAAGGGTCACTAGGACCAGGAAGATT
SNP primers for mapping cl1	SNPD07_15445591R	CTGTCAGATCCTCTTGTGTTGGGGAGAG
SSR primers for mapping cl1	DPL0061F	AGGTTCTCATGTCAACAAAGACAGT
SSR primers for mapping cl1	DPL0061R	TCATTTCTGCAACTTGTACTCACC
SSR primers for mapping cl1	NAU6430F	GAACAATTGTCGGGATTTTC
SSR primers for mapping cl1	NAU6430R	GAACAATTGTCGGGATTTTC
SSR primers for mapping cl1	DPL0897F	ATCGTACCCAATGGAGGATG
SSR primers for mapping cl1	DPL0897R	GCTTTCTTTCAGCTCCCTATTT
SSR primers for mapping cl1	NAU2974F	TGAACCTAAGCCCCATTATC
SSR primers for mapping cl1	NAU2974R	AGACACAAATACACTACTTCTCCA
primers for qRT-PCR GhSP	K9575F	GACAGACCCAGATGTTCCTGGTCCTAGTG
primers for qRT-PCR GhSP	K9575R	TTGCTTGAAGAGGAGGAACACAAACCTG
primers for qRT-PCR(detect the quality of cDNA)	Y347F	AGACCACCAAGTACTACTGCAC
primers for qRT-PCR(detect the quality of cDNA)	Y347R	CCACCAATCTTGTACACATCC
GhHIS3 (Reference gene as an internal standard)	Y8991F	CGGTGGTGTGAAGAAGCCTCAT
GhHIS3 (Reference gene as an internal standard)	Y8991R	AATTTCACGAACAAGCCTCTGGAA
primers for cloning gene GhSP(chr.D07)	K9051F	ATGGCAAAACTGTCAGATCCTCTTGTGT
primers for cloning gene GhSP(chr.D07)	K9051R	TTAGCGTCTTCTAGCAGCTGTTTCCCTTTG
primers for Virus-induced gene silencing assay (VIGS)	K7003F	gtgagtaaggttaccgaattcCCTTGGCATTTCGTAGTTCA
primers for Virus-induced gene silencing assay (VIGS)	K7003R	cgtgagctcggtaccggatccGAGTGATTGGGGATGTTATTGA
primers for test vector TRV1	TRV1F	TTGACTGATGTGCTGGGTTT
primers for test vector TRV1	TRV1R	CTTGGCTTTTACCTCGGATA
priners for test vector TRV2	TRV2F	TTGTTACTCAAGGAAGCACGAT
primers for test vector TRV2	TRV2R	TCCCCTATGGTAAGACAATGAG
Tissue expression analysis	GhSP-At-qRT-F	TACCTGAGGGAGCACTTACAC
Tissue expression analysis	GhSP-At-qRT-R	GAAGAGGAGGAACACAAACCTA
Tissue expression analysis	GhSP-Dt-qRT-F	TACCTGAGGGAGCACTTACAC
Tissue expression analysis	GhSPDAt-qRT-R	AAGAGGAGGAACACAAACCTG

Supplementary Table S3. Primers used for interaction of *GoSPs* with *FD in vivo* and detection of gene expression in wild-type and transgenic plants

**Purpose**	**Primer name**	**Sequence(5'-3')**
BD-GhSP vector	GhSP-F	ggggacaagtttgtacaaaaaagcaggctccATGGCAAAACTGTCAGAT
	GhSP-R	ggggaccactttgtacaagaaagctgggtcTTAGCGTCTTCTAGCAGC
BD-Ghsp vector	Ghsp-F	ggggacaagtttgtacaaaaaagcaggctccATGGCAAAACTGTCAGAT
	Ghsp-R	ggggaccactttgtacaagaaagctgggtcTTAGCGTCTTCTAGCAGC
BD-GbSP vector	GbSP-F	ggggacaagtttgtacaaaaaagcaggctccATGGCAAAACTGTCAGAT
	GbSP-R	ggggaccactttgtacaagaaagctgggtcTTAGCGTCTTCTAGCAGC
BD-Gbsp vector	Gbsp-F	ggggacaagtttgtacaaaaaagcaggctccATGGCAAAACTGTCAGAT
	Gbsp-R	ggggaccactttgtacaagaaagctgggtcTTAGCGTCTTCTAGCAGC
AD-GhFD vector	GhFD-F	ggggacaagtttgtacaaaaaagcaggctccATGTTATCACCATCCAATAAA
	GhFD-R	ggggaccactttgtacaagaaagctgggtcTTAAAATGGAGCTGTTGA
nYFP-GhSP vector	GhSP-F	ggggacaagtttgtacaaaaaagcaggctccATGGCAAAACTGTCAGAT
	GhSP-R	ggggaccactttgtacaagaaagctgggtcGCGTCTTCTAGCAGCTG
nYFP-Ghsp vector	Ghsp-F	ggggacaagtttgtacaaaaaagcaggctccATGGCAAAACTGTCAGAT
	Ghsp-R	ggggaccactttgtacaagaaagctgggtcGCGTCTTCTAGCAGCTG
nYFP-GbSP vector	GbSP-F	ggggacaagtttgtacaaaaaagcaggctccATGGCAAAACTGTCAGAT
	GbSP-R	ggggaccactttgtacaagaaagctgggtcGCGTCTTCTAGCAGCTG
nYFP-Gbsp vector	Gbsp-F	ggggacaagtttgtacaaaaaagcaggctccATGGCAAAACTGTCAGAT
	Gbsp-R	ggggaccactttgtacaagaaagctgggtcGCGTCTTCTAGCAGCTG
CYFP-GhFD vector	GhFD-F	ggggacaagtttgtacaaaaaagcaggctccATGTTATCACCATCCAATAAA
	GhFD-R	ggggaccactttgtacaagaaagctgggtcAAATGGAGCTGTTGATG
CLuc-GhSP vector	GhSP-F	ggggacaagtttgtacaaaaaagcaggctccATGGCAAAACTGTCAGAT
	GhSP-R	ggggaccactttgtacaagaaagctgggtcTTAGCGTCTTCTAGCAGC
CLuc-Ghsp vector	Ghsp-F	ggggacaagtttgtacaaaaaagcaggctccATGGCAAAACTGTCAGAT
	Ghsp-R	ggggaccactttgtacaagaaagctgggtcTTAGCGTCTTCTAGCAGC
CLuc-GbSP vector	GbSP-F	ggggacaagtttgtacaaaaaagcaggctccATGGCAAAACTGTCAGAT
	GbSP-R	ggggaccactttgtacaagaaagctgggtcTTAGCGTCTTCTAGCAGC
CLuc-Gbsp vector	Gbsp-F	ggggacaagtttgtacaaaaaagcaggctccATGGCAAAACTGTCAGAT
	Gbsp-R	ggggaccactttgtacaagaaagctgggtcTTAGCGTCTTCTAGCAGC
GhFD-NLuc vector	GhFD-F	ggggacaagtttgtacaaaaaagcaggctccATGTTATCACCATCCAATAAA
	GhFD-R	ggggaccactttgtacaagaaagctgggtcAAATGGAGCTGTTGATG
35S:GhSP vector	KpnI-GhSP-F	GGGGTACCATGGCAAAACTGTCAGATCCTC
	XbaI-GhSP-R	GCTCTAGATTAGCGTCTTCTAGCAGCTGTTT
35S:Ghsp vector	KpnI-Ghsp-F	GGGGTACCATGGCAAAACTGTCAGATCCTC
	XbaI-Ghsp-R	GCTCTAGATTAGCGTCTTCTAGCAGCTGTTT
35S:GbSP vector	KpnI-GbSP-F	GGGGTACCATGGCAAAACTGTCAGATCCTC
	XbaI-GbSP-R	GCTCTAGATTAGCGTCTTCTAGCAGCTGTTT
35S:Gbsp vector	KpnI-Gbsp-F	GGGGTACCATGGCAAAACTGTCAGATCCTC

	XbaI-Gbsp-R	GCTCTAGATTAGCGTCTTCTAGCAGCTGTTT
35S:GhSP-GFP vector	KpnI-GhSP-F	GGGGTACCATGGCAAAACTGTCAGATCCTC
	XbaI-GhSP-R	GCTCTAGAGCGTCTTCTAGCAGCTGTTT
35S:Ghsp-GFP vector	KpnI-Ghsp-F	GGGGTACCATGGCAAAACTGTCAGATCCTC
	XbaI-Ghsp-R	GCTCTAGAGCGTCTTCTAGCAGCTGTTT
35S:GbSP-GFP vector	KpnI-GbSP-F	GGGGTACCATGGCAAAACTGTCAGATCCTC
	XbaI-GbSP-R	GCTCTAGAGCGTCTTCTAGCAGCTGTTT
35S:Gbsp-GFP vector	KpnI-Gbsp-F	GGGGTACCATGGCAAAACTGTCAGATCCTC
	XbaI-Gbsp-R	GCTCTAGAGCGTCTTCTAGCAGCTGTTT
qRT-PCR	GhSP-qRT-F	TGAGGGAGCACTTACACTGG
	GhSP-qRT-R	CTCACTGTTTGCCTGCCTTT
qRT-PCR	Ghsp-qRT-F	TGAGGGAGCACTTACACTGG
	Ghsp-qRT-R	CTCACTGTTTGCCTGCCTTT
qRT-PCR	GbSP-qRT-F	TGAGGGAGCACTTACACTGG
	GbSP-qRT-R	CTCACTGTTTGCCTGCCTTT
qRT-PCR	Gbsp-qRT-F	TGAGGGAGCACTTACACTGG
	Gbsp-qRT-R	CTCACTGTTTGCCTGCCTTT
qRT-PCR	AtActin2-qRT-F	CTGGATCGGTGGTTCCATTC
	AtActin2-qRT-R	CCTGGACCTGCCTCATCATAC
qRT-PCR	AtFT-qRT-F	CCCACTGCAGGAATTCATCG
	AtFT-qRT-R	TTGTAGAAAACTGCGGCCAC
qRT-PCR	AP1-qRT-F	GCACCAAATCCAGCATCCTT
	AP1-qRT-R	CAGCCAAGGTTGCAGTTGTA
qRT-PCR	LFY-qRT-F	CGGAGTTAGGTTTTACGGCG
	LFY-qRT-R	GCGGAGAGTAGCAAATGACG
UBQ7(Reference gene)	UBQ7-F	AGAGGTCGAGTCTTCGGACA
	UBQ7-R	GCTTGATCTTCTTGGGCTTG

Supplementary Table S4. Segregation of cluster boll genes in cotton populations

Year	Population	Number with wild-type long branches	Number with axillary flowering and/or clustered bolls	χ^2 (Segregation ratio)
2014	(TM-1×Xinhai25) F_2	744	254	0.0855 (3:1)
2015	(TM-1×Xinhai25) BC_1	1305	1241	1. 5589 (1:1)
2016	(TM-1×Xinhai25) F_2	1339	411	2.0602 (3:1)
2014-15*	(TM-1×T582) F_2	281	99	0.2246 (3:1)
2014-15*	(TM-1×T582) BC_1	103	95	0.3232 (1:1)
2015	(TM-1×T582) F_2	1568	541	0.4781 (3:1)
2015	(TM-1×T582) BC_1	209	170	4.0132 (1:1)
2016	(TM-1×T582) F_2	1877	645	0.4446 (3:1)
2016	(TM-1×T582) BC_1	187	184	0.0242 (1:1)

* Scored in Sanya, Hainan Island, China.

Supplementary Table S5. Primers used in mapping and cloning of *GbAF* gene (*cl_1^b*)

Purpose	Primer names	Sequence(5'-3')
Indel primers for mapping GbAC	K3547F	GGAGAACCCCAAAGCTCACA
Indel primers for mapping GbAC	K3547R	GGTACTTGAAAAGCTCGCTGG
Indel primers for mapping GbAC	K3570F	TCGTTCACTAAGGGGTTCGC
Indel primers for mapping GbAC	K3570R	AGCGAGTCAATAGCATGCTGA
Indel primers for mapping GbAC	K3584F	TTGTGGGTGACAGGTGACAG
Indel primers for mapping GbAC	K3584R	TCCAAACCAATCAACTTTAATCTGT
Indel primers for mapping GbAC	K3602F	CGAGATGACACATGGAGGCA
Indel primers for mapping GbAC	K3602R	ATGGCCACTGCTACGTTCAA
Indel primers for mapping GbAC	K3612F	TTCGTCGGGGCGAATTTCAT
Indel primers for mapping GbAC	K3612R	ATGCGAACCCCTCAGTGAAT
Indel primers for mapping GbAC	K2928F	ACATGCTTGCATATTCCTTATTGT
Indel primers for mapping GbAC	K2928R	CACCAAAATCCCACGCCAAG
Indel primers for mapping GbAC	K2951F	TCGTCTGACTCCCTGTACTT
Indel primers for mapping GbAC	K2951R	TTGTTGAGAGAGATCAATTTGTTCA
Indel primers for mapping GbAC	K2975F	CCTTCTGAGAAACGATGCGC
Indel primers for mapping GbAC	K2975R	CGCCGGAAACTAGAGGAAGG
Indel primers for mapping GbAC	K2834F	AAAGAGGACAAGGCTACCGC
Indel primers for mapping GbAC	K2834R	ACCCAAGGGAGGATGAAACA
Indel primers for mapping GbAC	K2848F	TGCAAAATGAGCAACAAAAGCTG
Indel primers for mapping GbAC	K2848R	TGCACAAATAAAAGAGTAGTCAATCT
Indel primers for mapping GbAC	K2860F	AGGGTAGGGTTAGGTTAGGGT
Indel primers for mapping GbAC	K2860R	ACTTGATCCTACGAGTGCGA
Indel primers for mapping GbAC	K2868F	CCAGTTAGACTCCCAATTTGACG
Indel primers for mapping GbAC	K2868R	TCGTCCCCGATTTGCTCATT
Indel primers for mapping GbAC	K1602F	ATCTACCTTGCTGTGGTGCC
Indel primers for mapping GbAC	K1602R	GGGGCAAATACAAAAAGGGGT
Indel primers for mapping GbAC	K1796F	TTGCGATGTGGCGATGAGTA
Indel primers for mapping GbAC	K1796R	ACAGAGCAAAAACACAAAACACG
Indel primers for mapping GbAC	K1823F	GGAGAACCCCAAAGCTCACA
Indel primers for mapping GbAC	K1823R	ATGGCCACTGCTACGTTCAA
Indel primers for mapping GbAC	K1867F	TTAGGCGAGTCGAGTTGTGG
Indel primers for mapping GbAC	K1867R	AACTGTCGCTGTTCCACGAT
SSR primers for mapping GbAC	NAU2680F	TTTTTGGTCCTATTGGTGGT
SSR primers for mapping GbAC	NAU2680R	GCTTCAGGGTTTCTTGCTAA
SSR primers for mapping GbAC	NAU2734F	TTTTTGGTCCTATTGGTGGT
SSR primers for mapping GbAC	NAU2734R	ATGTTTCTGCAGGGACTTCT
SSR primers for mapping GbAC	NAU2974F	TGAACCTAAGCCCCATTATC
SSR primers for mapping GbAC	NAU2974R	AGACACAAATACACTACTTCTCCA
SNP primers for mapping GbAC	NAU300F	GAGAACTCGTTAAAGCACAATG
SNP primers for mapping GbAC	NAU300R	GTTAATAGAGTTGGGTTTCTCATG
SNP primers for mapping GbAC	NAU6772F	GAATTGAAGCAAACCTCATTAATTACC

SNP primers for mapping GbAC	NAU6772R	CTACCCTCATCTCATTCCAAAAAAC
SNP primers for mapping GbAC	NAU327F	CAACCATCACACCCAACAAA
SNP primers for mapping GbAC	NAU327R	AAAATGGAATGTTCCAGTCACC
SNP primers for mapping GbAC	NAU6664F	TTAATTTGTGACGCGAACTC
SNP primers for mapping GbAC	NAU6664R	CACCAACTCGATTACGAAAA
SNP primers for mapping GbAC	NAU4017F	AGTGAAGAAGCACCCAGAAC
SNP primers for mapping GbAC	NAU4017R	TCGGTATCCTCAAAACACCT
SNP primers for mapping GbAC	NAU6430F	GAACAATTGTCGGGATTTTC
SNP primers for mapping GbAC	NAU6430R	CTCACCGGCTCACTATATCC
SNP primers for mapping GbAC	NAU121F	TAGAGCCAAGTGGTGATCCC
SNP primers for mapping GbAC	NAU121R	AAAGGGGGGAATGATTATGC
SNP primers for mapping GbAC	NAU7933F	CTCTTGGGTCACCACCTGTT
SNP primers for mapping GbAC	NAU7933R	TGACGGAGGTCCTTCATTTC
SNP primers for mapping GbAC	NAU122F	ATCTCAGATTTAAACATATAATAGAGGG
SNP primers for mapping GbAC	NAU122R	TAAAATGAAGGCCATCAGGC
SNP primers for mapping GbAC	NAU5408F	CAAGACAGTGAAAAAGAAAAGG
SNP primers for mapping GbAC	NAU5408R	AGAGCAGCATCATCTGACAA
SNP primers for mapping GbAC	NAU2931F	CCGATTGGGAAACTAAGAAA
SNP primers for mapping GbAC	NAU2931R	CACCTTGTTGATGATGAGGA
SNP primers for mapping GbAC	NAU450F	CGACAGCGAGTGTGAAACAT
SNP primers for mapping GbAC	NAU450R	GGTGGGAAAAACGCAAACT
SNP primers for mapping GbAC	NAU2820F	TGCATCCTGAAGAAGAGACA
SNP primers for mapping GbAC	NAU2820R	GCCACCAATAAAGCAACTCT
SNP primers for mapping GbAC	NAU5120F	GCCACCAATAAAGCAACTCT
SNP primers for mapping GbAC	NAU5120R	TGCATCCTGAAGAAGAGACA
SNP primers for mapping GbAC	SNP07D15445079	GGAATACAAACCCGTGGATCCCTATGTTTTA
SNP primers for mapping GbAC	SNP07D15445079	GACTGTTAGGGTTCATAATTTCCTTGGCTTGATA
primers for qRT-PCR GbSP	K9576F	GTTACTAACAAGCCTAAGGTTGAGGTTA
primers for qRT-PCR GbSP	K9576R	GTGGCATCTGTTGTGCCGGGGATATCTG
primers for cloning gene GbSP (chr.D07)	K9052F	GAAAAAGAAAAGCAGCAGATAAGCATATCCA
primers for cloning gene GbSP (chr.D07)	K9052R	TTAGCGTCTTCTAGCAGCTGTTTCCCTTTGAG
primers for qRT-PCR (detect the quality of cDNA)	Y347F	AGACCACCAAGTACTACTGCAC
primers for qRT-PCR (detect the quality of cDNA)	Y347R	CCACCAATCTTGTACACATCC
GhHIS3 (Reference gene as an internal standard)	Y8991F	CGGTGGTGTGAAGAAGCCTCAT
GhHIS3 (Reference gene as an internal standard)	Y8991R	AATTTCACGAACAAGCCTCTGGAA
primers for cloning gene GhSP(chr.D07)	K9051F	ATGGCAAAACTGTCAGATCCTCTTGTGT

primers for cloning gene GhSP (chr.D07)	K9051R	TTAGCGTCTTCTAGCAGCTGTTTCCCTTTG
primers for Virus-induced gene silencing assay (VIGS)	K7003F	gtgagtaaggttaccgaattcCCTTGGCATTTCGTAGTTCA
primers for Virus-induced gene silencing assay (VIGS)	K7003R	cgtgagctcggtaccggatccGAGTGATTGGGGATGTTATTGA
primers for test vector TRV1	TRV1F	TTGACTGATGTGCTGGGTTT
primers for test vector TRV1	TRV1R	CTTGGCTTTTACCTCGGATA
primers for test vector TRV2	TRV2F	TTGTTACTCAAGGAAGCACGAT
primers for test vector TRV2	TRV2R	TCCCCTATGGTAAGACAATGAG

Supplementary Table S6. Candidate region for *cl_1* mutant loci identified by BSA-seq

Character	Candidate region				Key region 1				Key region 2			
	Chr.	Start (Mb)	End (Mb)	Length (Mb)	Chr.	Start (Mb)	End (Mb)	Length (Mb)	Chr.	Start (Mb)	End (Mb)	Length (Mb)
cluster boll(cl1)	D07	10.70	21.70	11.00	D07	14.42	14.65	0.24	D07	15.0	18.4	3.4
